# Targeted lipidomics meets transcriptomics: how cinobufagin rewires fatty acid, sphingolipid, and glycerophospholipid metabolism to combat hepatoma cell growth

**DOI:** 10.3389/fphar.2025.1664915

**Published:** 2026-02-09

**Authors:** Wanjun Shao, Congying Yu, Rufei Xu, Xiaoqian Rong, Ailin Yang

**Affiliations:** Binzhou Medical University, Yantai, China

**Keywords:** cinobufagin, fatty acid metabolism, hepatocellular carcinoma, integration of transcriptomics and metabolomics, sphingolipid metabolism, targeted lipid metabolomics

## Abstract

**Introduction:**

Hepatocellular carcinoma (HCC) is a common malignant tumor, is characterized by an early stage that is not easy to diagnose and a high mortality rate in the late stage, which is a serious threat to patients' lives. Abnormalities in lipid metabolism are closely related to the development of HCC. Integrating transcriptomics and metabolomics analyses can help in the study of drug mechanism of action. Cinobufagin, is the main active ingredient for chinese medicine Chansu to exert anti-tumor effects, but the effects of cinobufagin on abnormal lipid metabolism in tumor cells are not clear.

**Methods:**

We employed targeted lipid metabolomics to profile alterations in key lipid classes. Furthermore, integrated transcriptomics and metabolomics analyses were conducted to identify critical pathways involved in cinobufagin's action.

**Results:**

In this study, we demonstrate through the results of targeted lipid metabolomics that cinobufagin interferes with fatty acyls, sphingolipids, glycerophospholipids, glycerolipids, saccharolipids, and sterol lipids. The results of integration of transcriptomics and metabolomics identified that intervention in fatty acid metabolism (including biosynthesis of unsaturated fatty acids, fatty acid biosynthesis, fatty acid degradation, and fatty acid elongation), sphingolipid metabolism (including sphingolipid metabolism, glycosphingolipid biosynthesis-globo and isoglobo series, glycosphingolipid biosynthesis-lacto and neolacto series, glycosphingolipid biosynthesis-ganglio series), and glycerophospholipid metabolism (including glycerophospholipid metabolism, ether lipid metabolism, glycosylphosphatidylinositol (GPI)-anchor biosynthesis) may be partially responsible for the effect of anti-hepatoma cell growth induced by cinobufagin.

**Discussion:**

Our findings demonstrate that cinobufagin exerts anti-HCC activity partially through lipid metabolism, particularly by targeting fatty acid, sphingolipid, and glycerophospholipid pathways. This study is of great significance for the application of cinobufagin and chansu in clinical HCC treatment and promotes the development of new drugs from traditional Chinese medicine in the field of antitumor.

## 1 Introduction

Hepatocellular carcinoma (HCC), as a common malignant tumor in clinical practice, is characterized by an early stage that is not easy to diagnose and a high mortality rate in the late stage (Rumgay et al., 2022), which is a serious threat to patients' lives. Hepatitis B and C viruses, fatty liver, alcohol-related cirrhosis, smoking, obesity, diabetes are a few of risk factors (Center and Jemal, 2011).At present, surgery remains the efficacious methods in the treatment of liver cancer. Although it can inhibit the development of HCC, it also causes damage to the organism, and the treatment effect is often poor. Compared with western drugs, traditional chinese medicine has the advantages of multi-target, multi-level, multi-pathway, and integrated regulation (Zhang and Xiao, 2021). Currently, traditional chinese medicine and its related active components are being more widely applied in clinical cancer treatment (Li JJ. et al., 2021).

Lipids play important roles in cell function (Wu et al., 2023), and lipid metabolism is central to life maintenance (Lemmon, 2008). Under normal conditions, the liver synthesizes cholesterol and triglycerides, packages them into lipoproteins, and secretes them into the bloodstream to regulate the body's energy supply and metabolism. To ensure cellular survival, lipid homeostasis allows rapid adaptation to metabolic shifts through integrative systems. Under conditions of energy deficiency or nutrient depletion, metabolic intermediates are desperately needed by cells for energy production and nutrition synthesis (Alannan et al., 2020). Therefore, the function of lipids becomes particularly important and cannot be ignored in cancer and its related diseases. Lipids are mainly processed in the liver, which ensures stable lipid and lipoprotein metabolism under normal physiological conditions, and lipids also play an important role in the physiological functioning of the liver, as well as influencing the pathological progression of liver diseases. As a result, hepatocellular damage impairs liver function and may alter lipid metabolism, which, thus, might aid in the development and progression of liver tumors (Du et al., 2022). Further, lipids are also involved in cell signaling. During cancer development, cancer cells increase adipogenesis, fatty acid uptake and fatty acid oxidation for energy production and lipid accumulation (Martin-Perez et al., 2022). The typical rationale for elevated lipid metabolism in cancer cells is that increased lipids are required for plasma membrane synthesis and energy production. Abnormal lipid metabolism is strongly associated with the development of HCC.

Chansu is the dried secretion of the post-auricular and skin glands of *Bufo bufo gargarizans* Cantor and *Bufo melanostictus* Schneider. Clinical studies have demonstrated the effectiveness of chansu in the treatment of HCC and hepatitis. Cinobufagin, a bufadienolide is the main active ingredient for chansu to exert anti-tumor effects. Studies have shown that cinobufagin displays good anti-tumor activity against HCC, breast cancer, and colon cancer. In our previous study, we have revealed that cinobufagin reduces the proliferation and colony forming ability of human hepatoma cells *in vitro*, and in addition, induces mitotic arrest and DNA damage in cancer cells (Yang et al., 2022). Network pharmacology analysis has demonstrated that the inhibitory effect of cinobufagin on HCC cells involves several signaling pathways, such as proteoglycans in cancer, HIF-1 signaling pathway, VEGF signaling pathway, ErbB signaling pathway, and PI3K-AKT signaling pathway (Yang et al., 2021). Cinobufagin exhibits excellent anti-tumor effects in liver cancer cells by inhibiting the Aurora Kinase A (AURKA)-mTOR-eucalyptic translation initiation factor 4E (eIF4E) axis (Jin et al., 2020). Cinobufagin activates caspase family and PARP-mediated apoptosis by up-regulating Bcl2 associated X (BAX) expression and down-regulating Bcl2 apoptosis regulator (BCL-2) expression (Qi et al., 2011). Cinobufagin reduces neovascularization and triggers endothelial cell apoptosis by inducing reactive oxygen species (ROS) accumulation and mitochondrial dysfunction (Li C. et al., 2019). However, there are few studies on the molecular mechanisms of its anti-liver cancer effect by intervening lipid metabolism.

In this study, analysis of differential lipid compounds in response to cinobufagin treatment based on targeted lipid metabolomics shows that six classes of differentially regulated lipid compounds, including fatty acyls, sphingolipids, glycerophospholipids, glycerolipids, saccharolipids, sterol lipids. After administration of cinobufagin, the lipids that are upregulated include all identified monosialodihexosylgangliosides (GM3), hexosylceramide (HexCer), hemibiosmonacylglycerophosphate (HBMP), cholesterol esters (CE), and triacylglycerol (TAG), while the lipids that are downregulated include all identified acylcarnitine (Acar) and cardiolipin (CL). In addition, cinobufagin upregulates most of identified ceramides (Cer), sphingomyelins (SM), and diacylglycerols (DAG), while downregulates most of identified free fatty acids (FA) and phosphatidylethanolamines (PE). Subsequently, through metabolomics combined with transcriptomics analysis, several related metabolic pathways are identified, including fatty acid metabolism (including biosynthesis of unsaturated fatty acids, fatty acid biosynthesis, fatty acid degradation, and fatty acid elongation), sphingolipid metabolism (including sphingolipid metabolism, glycosphingolipid biosynthesis-globo and isoglobo series, glycosphingolipid biosynthesis-lacto and neolacto series, glycosphingolipid biosynthesis-ganglio series), and glycerophospholipid metabolism (including glycerophospholipid metabolism, ether lipid metabolism, glycosylphosphatidylinositol (GPI)-anchor biosynthesis), which may be responsible for the anti-tumor effect of cinobufagin. This study provides new theoretical basis for the clinical application of cinobufagin and chansu, and promotes the development and utilization of active ingredients from traditional chinese medicine.

## 2 Materials and methods

### 2.1 Reagents

DMEM medium, fetal bovine serum (FBS), penicillin-streptomycin, 0.25% Trypsin-EDTA solution, and PBS buffer were purchased from Corning Life Sciences (Steuben County, New York, USA).

### 2.2 Cell culture

Human hepatoma HepG2 cell line was cultured in DMEM medium containing 10% fetal bovine serum and 1% penicillin-streptomycin in a cell culture incubator at a temperature of 37 °C with 5% CO_2_.

### 2.3 Extraction of metabolites from cell samples

Cinobufagin (purity: 99.6%) was purchased from National Institutes for Food and Drug Control (Beijing, China), it was dissolved in DMSO to get 10 mM stock solution, and then diluted to the working concentration for subsequent experiments. The structure of cinobufagin can be found in our previous article (Yang et al., 2021). HepG2 cells in the drug administration group were treated with cinobufagin (1 μM) for 24 h and the control group was cultured with complete DMEM medium. After drug treatment, an equal number of cells were digested and collected. Methanol (0.75 mL) was added to sample, which was placed into a glass tube with a Teflon lined cap, and the tube was vortexed. 2.5 mL of MTBE was added and the mixture was incubated for 1 h at room temperature in a shaker. Phase separation was induced by adding 0.625 mL of MS-grade water. Upon 10 min of incubation at room temperature, the sample was centrifuged at 1,000 *g* for 10 min. The upper (organic) phase was collected, and the lower phase was re-extracted with 1 mL of the solvent mixture (MTBE/methanol/water (10:3:2.5, v/v/v)), and collecting the upper phase. Combined organicphases were dried and dissolved in 100 μL of isopropanol for storage. Then analyzed by LC-MS/MS.

### 2.4 UHPLC-MS/MS analysis

Novogene Co., Ltd. (Beijing, China) employed a Vanquish UHPLC system (Thermo Fisher, Germany) in conjunction with an Orbitrap Q Exactive™ HF mass spectrometer (Thermo Fisher, Germany) to conduct UHPLC-MS/MS analysis. The chromatographic separation was performed on a Thermo Accucore C30 column (150 × 2.1 mm, 2.6 μm) with the mobile phase of 10 mM ammonium acetate and 0.1% formic acid in acetonitrile/water (6/4) (A) and 10 mM ammonium acetate and 0.1% formic acid in acetonitrile/isopropanol (1/9) (B) at a flow rate of 0.35 mL/min. The elution gradient configuration was as follows: 30% B, initial; 30% B, 2 min; 43% B, 5 min; 55% B, 5.1 min; 70% B, 11 min; 99% B, 16 min; 30% B, 18.1 min. Q Exactive™ HF mass spectrometer was operated in positive [negative] polarity mode with sheath gas: 40 psi, sweep gas: 0 L/min, auxiliary gasrate: 10 L/min [7 L/min], spray voltage: 3.5 kV, capillary temperature: 320 °C, heater temperature: 350 °C, S-Lens RF level: 50, scan range: 114–1700 m/z, automatic gain control target: 3e6, normalized collision energy: 22 eV; 24 eV; 28 eV [22 eV; 24 eV; 28 eV], Injection time: 100 m, Isolation window: 1 m/z, automatic gaincontrol target (MS^2^): 2e5, dynamic exclusion: 6 s.

The Compound Discoverer 3.01 (CD3.1, Thermo Fisher) was used to process the raw data files produced by the UHPLC-MS/MS to perform peak alignment, peak selection, and quantification for every metabolite. The tolerances for retention times, actual masses, signal intensities, signal/noise ratios, and minimum intensity were set at 0.2 min, 5 ppm, 30%, and 100,000, respectively. After that, peak intensities were normalized to the total spectral intensity. The normalized data was used to predict the molecular formula based on additive ions, molecular ion peaks and fragment ions. And then peaks were matched with the Lipidmaps and Lipidblast database to obtained the accurate qualitative and relative quantitative results. Statistical analyses were performed using the statistical software R (R version R-3.4.3), Python (Python 2.7.6 version) and CentOS (CentOS release 6.6), when data were not normally distributed, normal transformations were attempted using of area normalization method.

### 2.5 Quality control

#### 2.5.1 QC sample correlation analysis

Based on the relative quantitative values of lipid compounds to calculate the pearson correlation coefficient between QC samples (Rao et al., 2016), the higher the correlation between QC samples (*R* ^2^ closer to 1) indicates that the whole process of detection is more stable, and the data quality is higher.

#### 2.5.2 Total principal components analysis (PCA)

The data was log-transformed and standardized using MetaX software (Wen et al., 2017). The peaks extracted from all experimental samples and QC samples were analyzed by PCA, and the smaller the difference between the QC samples, the better the stability of the whole method and the higher the quality of the data.

### 2.6 Differential lipid compound screening

#### 2.6.1 Principal components analysis (PCA)

The multidimensional data was subjected to dimensionality reduction and regression analysis on the basis of maximizing the retention of the original information, followed by screening of differential metabolites and subsequent analysis. PCA was used to observe the overall distribution trend between the cinobufagin-treated group and control group.

#### 2.6.2 Partial least squares discriminant analysis (PLS-DA)

Partial Least Squares Discriminant Analysis (PLS-DA) is a supervised statistical method for discriminant analysis (Boulesteix and Strimmer, 2007). To predict the sample category, the technique models the link between metabolite expression and sample category using partial least squares regression. After establishing the PLS-DA model for each comparison group, the model evaluation parameters (R2, Q2) were obtained using 7-fold cross-validation (seven cycles of cross-validation). The closer R2 and Q2 are to 1, the more stable and reliable the model is.

The model is also be sorted to see if it is overfitting to assess the model’s quality. If the model is not overfitting, it can better represent the sample and serve as a prerequisite for identifying biomarkers. “Overfitting” indicates that the model is unsuitable for describing the sample and should not be used for subsequent analysis. The specific method involves randomly shuffling the group labels of each sample before modeling and prediction. Each modeling process corresponds to a pair of R2 and Q2 values. Based on 200 iterations of shuffling and modeling, regression lines for Q2 and R2 values can be obtained. If the R2 value is greater than the Q2 value and the Q2 regression line intercepts the Y-axis at less than zero, it indicates that the model has not “overfitted”.

#### 2.6.3 Differential lipid compound screening

The screening of differential lipid compounds mainly referred to the three parameters of VIP, FC, and *P*-value. VIP refers to the Variable Importance in the Projection of the first principal component of the PLS-DA model, and the VIP value indicates the contribution of metabolites to the subgroups. FC refers to the fold change, which is the ratio of the mean quantification values of each metabolite across all biological replicates in the comparison group. And the *P*-value is calculated by the T-test, which indicates the level of significance of the difference. Differential lipid compounds were screened by setting thresholds of VIP >1.0, FC > 1.5 or FC < 0.667 and *P*-value <0.05.

### 2.7 Transcriptome sequencing

Total RNA was extracted from HepG2 cells, treated with or without 1 μM cinobufagin for 24 h, using the E.Z.N.A.® Total RNA Kit I. RNA Integrity Number (RIN) ≥ 8.0 and 28S/18S rRNA ratio ≥1.8. The RNA samples were then subjected to sequencing on the BGISEQ-500 platform by Beijing Genomics Institute (BGI, Shenzhen, China).

This project followed a standard RNA-seq analytical pipeline. Firstly, raw sequencing data generated on the BGISEQ platform underwent rigorous quality control, which involved the removal of adapter-contaminated and low-quality reads to obtain high-quality clean reads. Subsequently, these clean reads were aligned to the reference genome sequence using HISAT, achieving an average alignment rate of 93.41%. The clean reads were also aligned to the reference gene set using Bowtie2, with an average alignment rate of 84.87%. Based on these alignments, expression quantification was performed for each gene, leading to the detection of 14,905 genes in total. In this study, biological replicates are grouped together for comparative analysis, a differential expression analysis between the control and cinobufagin-treated groups identified 6,900 significantly differentially expressed genes. Differentially expressed genes (DEGs) were identified with a threshold of |log2FC| ≥ 1 and FDR ≤0.001. We calculated *P* values using phyper. Q value (corrected *P* value) ≤ 0.05 was used as the threshold (Yang et al., 2023).

### 2.8 Statistical analysis

Using the software GraphPad Prism 10, the two-tailed Student’s t-test was used to determine the significance of the difference between the groups. Data is expressed as mean ± SEM. *P* < 0.05 was considered to be statistically significant.

## 3 Results

### 3.1 Data quality control results

#### 3.1.1 QC sample correlation analysis results

**FIGURE 1:**
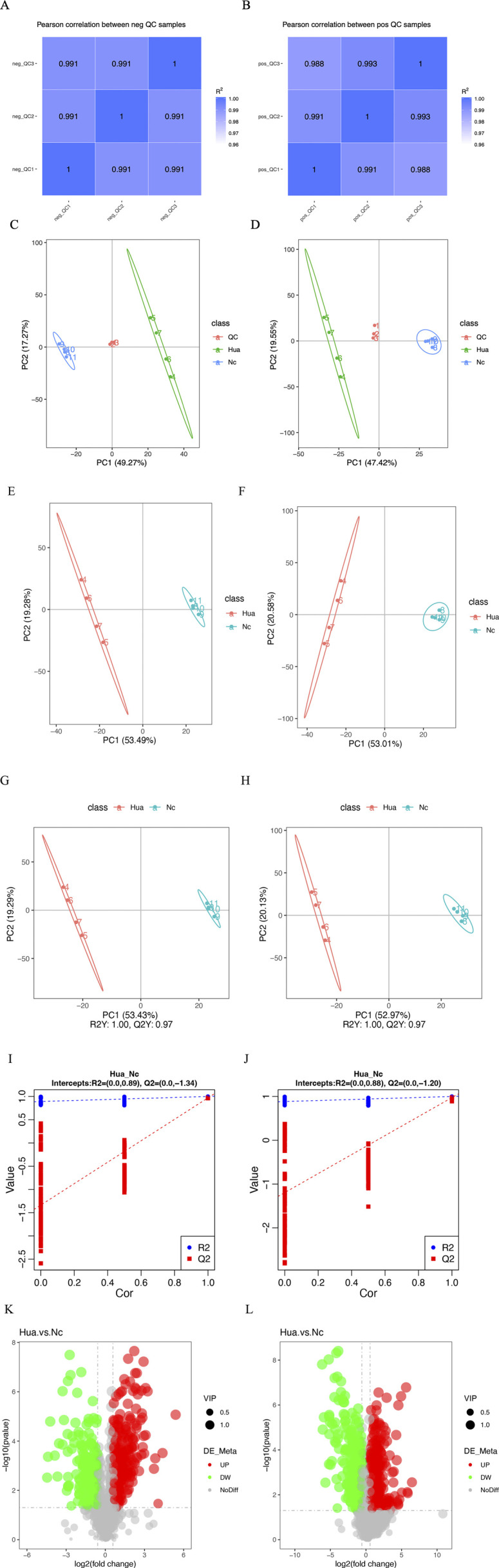
Metabolomics characteristics of human hepatoma cells treated with or without cinobufagin. **(A,B)** QC sample correlation analysis results of the (LC-MS). **(A)** negative mode, **(B)** positive mode. **(C,D)** Total principal components analysis (PCA) of the metabolism differences between tumor cells treated with or without cinobufagin based on liquid chromatography mass spectrometry (LC-MS). **(C)** negative mode, **(D)** positive mode. **(E,F)** Differential lipid principal components analysis (PCA) of the metabolism differences between tumor cells treated with or without cinobufagin based on liquid chromatography mass spectrometry (LC-MS). **(E)** negative mode, **(F)** positive mode. **(G,H)** Partial least squares discriminant analysis (PLS-DA) of the metabolism differences between tumor cells treated with or without cinobufagin based on LC-MS.**(G)** negative mode, **(H)** positive mode. **(I)** The results of permutation test of negative mode. **(J)** The results of permutation test of positive mode. **(K, L)** Volcano Plots of cinobufagin-treated group/control group. **(K)** negative mode, (L) positive mode.

The correlation coefficients between the QC samples of the experimental results, both in the positive and negative ion modes, were above 0.9, indicating that the experiment was well stabilized throughout the detection process and that the data were of high quality (Figures 1A,B).

#### 3.1.2 Total principal components analysis (PCA) results

By PCA analysis of the total sample (Figures 1C,D), the sample points were close to each other with a high degree of aggregation, and there was a high degree of dispersion between the sample points of the control group and the drug administration group, the results showed that the sample quality was good enough to be analyzed in the next step.

### 3.2 Differential lipid compound screening results

#### 3.2.1 Principal components analysis (PCA) results

As shown in Figures 1E,F, 53.49% of the metabolites in the first principal component in the negative ion mode were able to distinguish the two groups of samples, while 19.28% of the metabolites in the second principal component were able to differentiate between the two sample groups. The results of principal component analysis further showed that the two groups of samples were in different regions in the axes with a large degree of dispersion, indicating significant lipid differences between the control group and cinobufagin-treated group. The similar results were also identified in the positive ion mode.

#### 3.2.2 Results of the partial least squares discriminant (PLS-DA) analysis

This analysis realizes the prediction of the sample categories. There was no overlap between the two samples, indicating significant metabolite differences between the two groups of samples. As shown in Figures 1G,H, 53.43% of the metabolites in the first principal component in the negative ion mode were able to distinguish the two groups of samples, while 19.29% of the metabolites in the second principal component were able to distinguish the two groups of samples. Furthermore, Q2 and R2 were around 1, suggesting that the model was dependable and steady. In the positive ion model, 52.97% of the metabolites in the first principal component can distinguish the two groups of samples, while 20.13% of the metabolites in the second principal component can distinguish the two groups of samples. Furthermore, R2 and Q2 were around 1. The model was trustworthy and stable.

#### 3.2.3 Permutation test results

The model was ranked and validated, and it was found that the model was appropriately constructed because it was not “overfitted”. As shown in figure Figures 1I,J, R2Y is larger than Q2Y, which means that the samples were better described by the model.

#### 3.2.4 Differential lipid compound screening results

**FIGURE 2:**
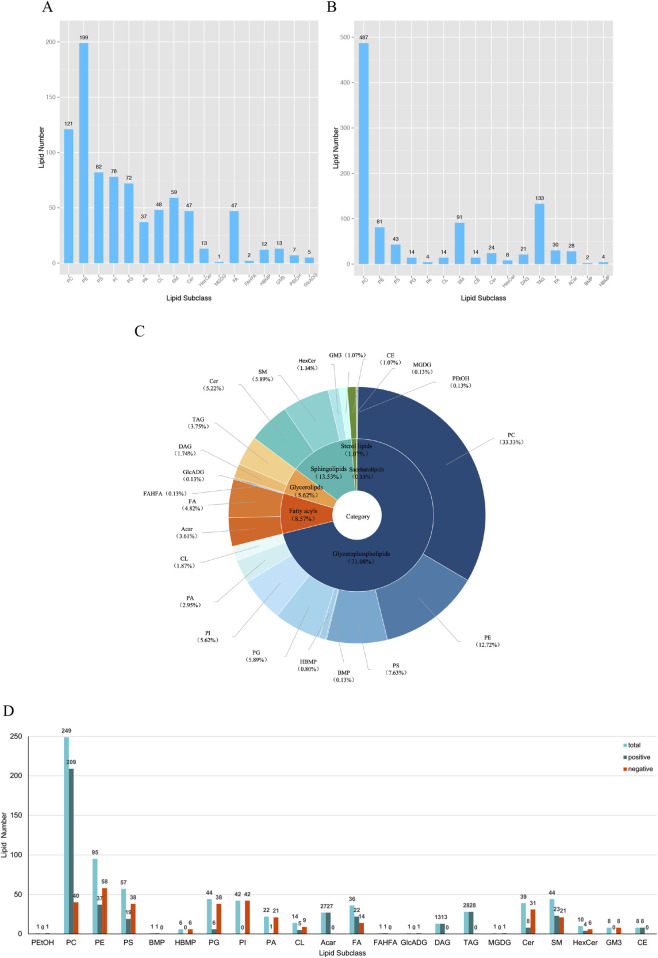
Specific number and subclass of various lipids. **(A)** Total number of various lipids screened in negative ion mode. phosphatidylcholines (PC), phosphatidylethanolamines (PE), phosphatidylserines (PS), phosphatidylinositols (PI), phosphatidylglycerols (PG), phosphatidic acids (PA), cardiolipin (CL), sphingomyelines (SM), ceramides (Cer), hexosyl ceramide (HexCer), cholesteryl esters (CE), monogalactosyldiacylglycerols (MGDG), free fatty acid (FA), fatty acid ester of hydroxyl fatty acid (FAHFA), hemibismonoacylglycerophosphate (HBMP), bis(monoacylglycero)phosphates (BMP), monosialodihexosylganglioside (GM3), phosphatidylethanol (PEtOH), glucuronosyldiacylglycerol (GlcADG), diacylglycerols (DAG), triacylglycerols (TAG), acylcarnitine (Acar). **(B)** Total number of various lipids screened in positive ion mode. **(C)** Percentage of distribution of all differential lipids. **(D)** Bar graph of the number of all differential lipids in positive and negative ion mode.

The volcano plots (Figures 1K,L) visualize the number and degree of differential lipid compound, which green represents downregulated differential lipid metabolites and red represents upregulated differential lipid metabolites. The total number of identified metabolites in the positive ion mode was 1,553, and the total number of significantly differentiated metabolites was 628. The total number of identified metabolites in the negative ion mode was 1,206, and the total number of significantly differentiated metabolites was 468. The International Lipid Classification and Nomenclature Committee (ILCNC) classifies lipid compounds into eight major types, and various lipid molecules regulate processes such as cell proliferation, apoptosis, and drug response. We first showed the bar charts of the specific total number of various lipids in the positive and negative ion mode (Figures 2A,B). Because we analyzed the differential lipids among them, we performed a summary analysis of the classes and subclasses of filtered differential lipid compound (Table 1). The pie charts and bar charts showed the changes in the number and percentage of various differential lipids (Figures 2C,D).

**TABLE 1:** The classes and subclasses of filtered differential lipid compound.

Class	subClass	Number
Glycerophospholipids	Phosphatidylethanol (PEtOH)	1
	Phosphatidylcholine (PC)	249
	Phosphatidylethanolamine (PE)	95
	Phosphatidylserine (PS)	57
	Bis(monoacylglycero)phosphate (BMP)	1
	Hemibismonoacylglycerophosphate (HBMP)	6
	Phosphatidylglycerol (PG)	44
	Phosphatidylinositol (PI)	42
	Phosphatidic acid (PA)	22
	Cardiolipin (CL)	14
Glycerolipids	Diacylglycerol (DAG)	13
	Triacylglycerol (TAG)	28
	Glucuronosyldiacylglycerol (GlcADG)	1
Fatty acyls	Acylcarnitine (Acar)	27
	Free fatty acid (FA)	36
	Fatty acid ester of hydroxyl fatty acid (FAHFA)	1
Saccharolipids	Monogalactosyldiacylglycerol (MGDG)	1
Sphingolipids	Ceramide (Cer)	39
	Sphingomyeline (SM)	44
	Hexosyl ceramide (HexCer)	10
	Monosialodihexosylganglioside (GM3)	8
Sterol lipids	Cholesteryl ester (CE)	8

### 3.3 Analysis of differential lipid compounds

#### 3.3.1 Fatty acyls

**FIGURE 3:**
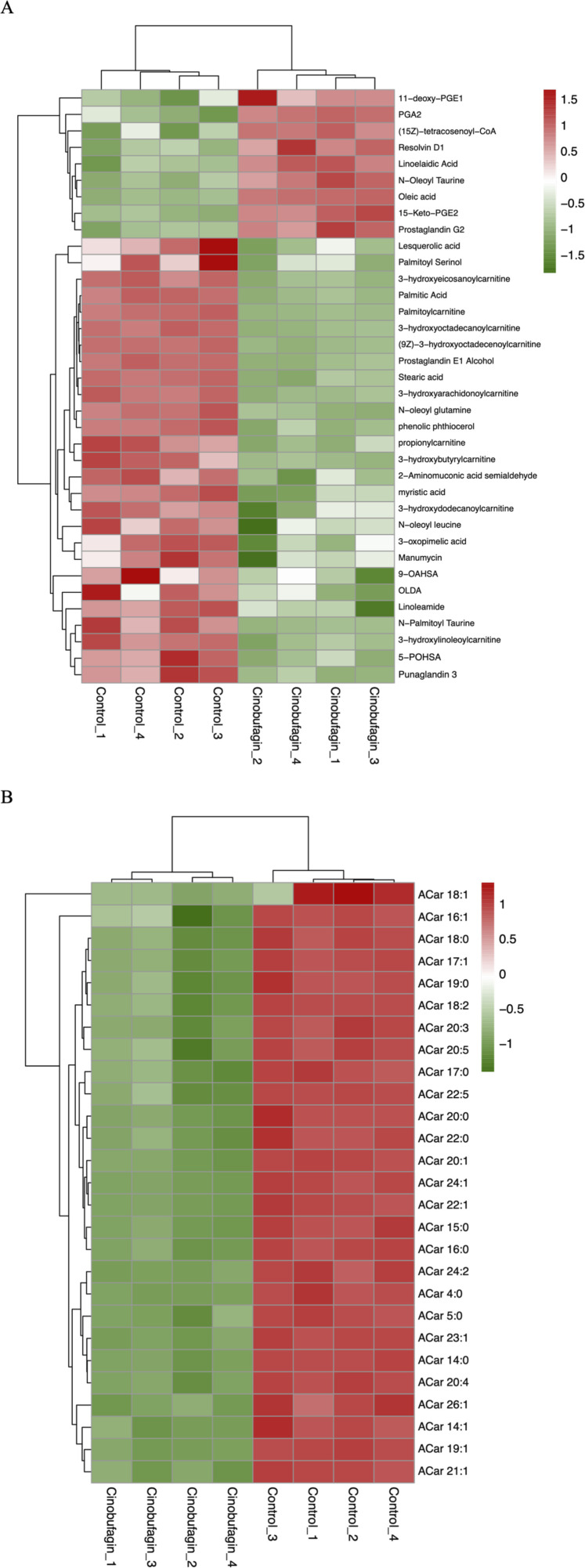
Heatmap analysis of the fatty acyls in human hepatoma cells treated with or without cinobufagin. **(A)** Heatmap analysis of the identified FA in HepG2 cells treated with or without cinobufagin. **(B)** Heatmap analysis of the identified Acar in HepG2 cells treated with or without cinobufagin. Red represents high level and green represents low level. Each row represents a single metabolite, each column represents one sample.

Numerous studies have shown that free fatty acid (FA) and acylcarnitine (Acar) play an important role in the development of cancer. The serum levels of various FAs and Acars fluctuate as the cancer course progresses. Changes in the levels of FA-related and Acar-related differential lipid compounds in response to cinobufagin were compared by heatmap, the levels of 9 FAs levels were increased and 27 FAs were decreased. The levels of all 27 identified Acars were decreased. These results suggest that cinobufagin has a significant effect on FAs and an obvious inhibitory effect on Acars in human HCC cells (Figures 3A,B).

As shown in Figure 3A, the saturated FAs palmitic acid, stearic acid, and myristic acid were decreased after cinobufagin administration. A prospective study has shown a positive correlation between saturated FA and HCC risk. In a published report, a combined transcriptomics and metabolomics approach is used to identify palmitic acid as a marker of HCC progression and poor patient survival (Beyogl et al., 2013). Studies has demonstrated that palmitic acid can promote the development of HCC by disrupting SIRT1-mediated deacetylation and increasing the levels of acetylated *α*-fetoprotein (AFP) (Xue et al., 2020). In terms of diet, palmitic acid-rich diets reprogram cancer cell metabolism and promote tumor innervation and metastasis formation (Pascual et al., 2021). In contrast, palmitic acid can be reduced by exercise to improve the tumor microenvironment (Zhang et al., 2024). Studies have identified the catalytic activity of stearic acid and its monoacylglycerol precursor are also enhanced in HCC tumors (Beyogl et al., 2013). The addition of myristic acid also leads to an increase in ACOT8-mediated growth of HCC cells (Hung et al., 2014).

In contrast, whereas unsaturated fatty acids oleic acid, linolenic acid, Resolvin D1 (RvD1), N-acyl taurines, PGA2, and PGE2 were elevated in response to cinobufagin. Previous research has shown that oleic acid (OA) inhibits proliferation and induces apoptosis of cancer cells (Giulitti et al., 2021). Resolvin D1, as a derivative of the n-3 polyunsaturated fatty acid DHA (Docosahexaenoic Acid), contains eicosapentaenoic acid (EPA) or eicosahexaenoic acid (DHA) found in fish oils, and it is an endogenous lipid mediator with anti-inflammatory and anticancer properties. It has been shown that RvD1 inhibits EMT and cancer stem cells in HCC by targeting FPR2/ROS/FOXM1 signaling, thereby impairing secretion of CAFs-derived cartilage oligomeric matrix protein (COMP) (Sun et al., 2019). Other studies have shown that cancer therapy reduces tumor burden by killing tumor cells, but it simultaneously generates tumor cell debris that may stimulate inflammation and tumor growth. In contrast, RvD1 is mediator that inhibits debris-stimulated cancer progression through endogenous clearance of tumor cell debris by macrophage phagocytosis (Sulciner et al., 2018). RvD1 inhibits inflammatory cytokine secretion and NF-κB/AP-1 activity, preventing concanavalin A-induced liver damage and hepatitis that progressed to HCC in mice (Kuang et al., 2016). Studies have identified that linolenic acid induces apoptosis in cancer cells through p53 and caspase activation (Dutta et al., 2023). N-Oleoyl taurine belongs to a group of N-acyl taurines (N-Oleoyl taurine), it has been shown that the addition of N-Oleoyl taurine to cancer cell cultures results in reduced proliferation (Chatzakos et al., 2012). In human non-small cell lung cancer cells, Prostaglandin A2 (PGA2) inhibits the expression of cyclin D1 via reducing the stability of cyclin D1 mRNA, which has anticancer effects. Reports have confirmed that 5-keto prostaglandin E2 (15-keto PGE2), which is produced when PGE2 is oxidized, has anti-inflammatory and anticancer properties. STAT3 phosphorylation was inhibited in xenograft tumor by subcutaneous injection of 15-keto PGE2 (Lee et al., 2019).

All identified Acar levels were decreased in cancer cells treated with cinobufagin. The organ principally involved in the synthesis and metabolism of endogenous carnitine is the liver. Abnormalities in the metabolism of acylcarnitine are linked to the stage of liver disease. Generally speaking, as the illness progresses, abnormalities in the metabolism of acylcarnitine get worse. Several studies have shown that butylcarnitine levels are significantly elevated in patients with nonalcoholic fatty liver disease (NAFLD). Free carnitine, propionylcarnitine, butylcarnitine, and 2-methylbutylcarnitine are significantly increased as the disease progresses to the more severe form of NASH (Li S. F. et al., 2019). An important feature of HCC is the significant change in large amounts of Acylcarnitine (Acars). Studies have found that long-chain Acars (>C14) accumulate in HCC tumors (Huang et al., 2013). Comparing patients diagnosed with HCC to the chronic HCV infection, higher levels of short-chain (Acar 2:0, 3:0, 5:1) and long-chain (Acar 14:1, 16:2, and 18:1) are observed (Caponigro et al., 2023). Patients with NAFLD-driven liver fibrosis have been shown to have elevated levels of long-chain (Acar 14:1 and 18:1) as well as increased rates of HCC development (Enooku et al., 2019). Acar 18:1, a long-chain species of Acars, functions as a carcinometabolite by triggering transcriptional activators and signal transducers, which endows HCC cells with stem cell characteristics (Fujiwara et al., 2018). Fatty acid *β*-oxidation may affect the progression of HCC. The primary function of Acars is to transport fatty acids into the mitochondrial matrix for *β*-oxidation. *β*-oxidation of long-chain fatty acids has been reported to be an important source of energy production in cancer cells. The increase in long-chain Acars in cancer tissues suggests that more fatty acids enter the mitochondria to provide energy (Lu et al., 2016). The reduction of Acars levels in liver cancer cells may be responsible for the anti-hepatoma effects of cinobufagin. In summary, these results provide clues for analyzing the role of fatty acyls in cancer.

#### 3.3.2 Sphingolipids

**FIGURE 4:**
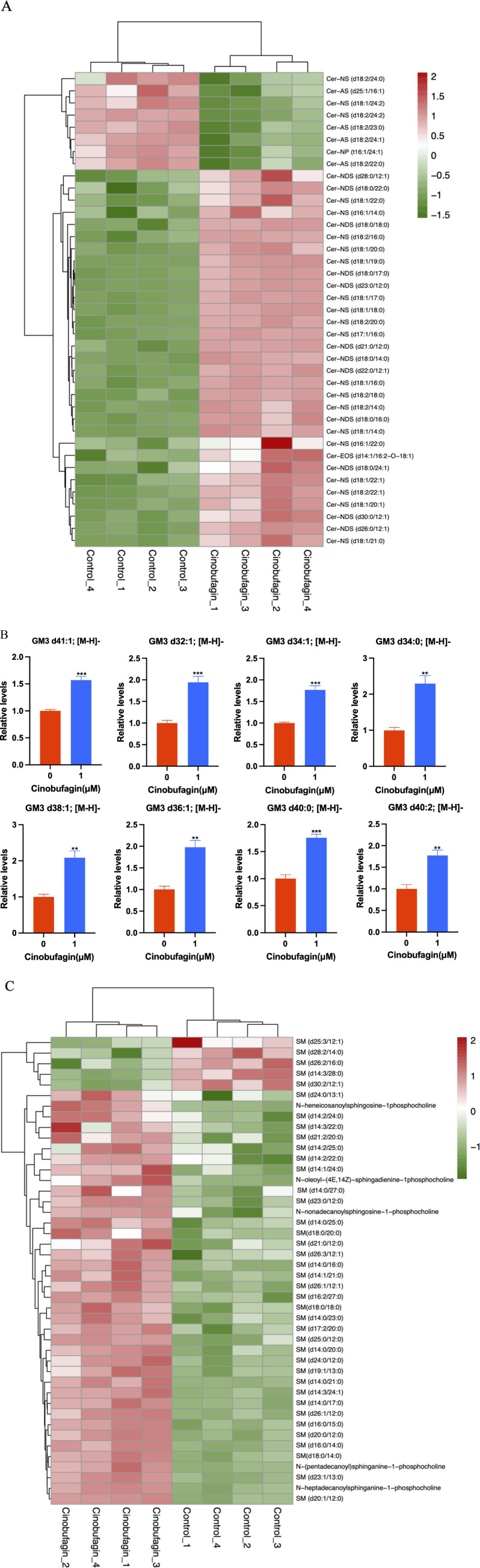
Graph analysis of the identified Sphingolipids in HepG2 cells treated with or without cinobufagin. **(A)** Heatmap analysis of the identified Cer in HepG2 cells treated with or without cinobufagin. **(B)** Relative levels of the GM3 in HepG2 cells treated with or without cinobufagin. **P < 0.01, ***P < 0.001. **(C)** Heatmap analysis of the identified SM in HepG2 cells treated with or without cinobufagin. Red represents high level and green represents low level. Each row represents a single metabolite, each column represents one sample.

In our study, after cinobufagin treatment, the levels of 39 sphingomyelins (SM) were increased and 5 SMs were decreased, the levels of 31 ceramides (Cer) were increased and eight ceramides (Cer) were decreased, the levels of all eight identified monosialodihexosylgangliosides (GM3) were increased, and the levels of all eight identified hexosyl ceramide (HexCer) contents were all increased. The results showed that cinobufagin had significant effects on sphingolipid levels including Cer, SM, GM3, and HexCer in human HCC cells (Figure 4).

Because sphingolipids (SPLs) are an essential part of the plasma membrane and contribute to the lipid raft composition that fuels proto-oncogenic signaling, they are relevant to the development of tumors. Cer serves as the key mediator for a vast number of SPLs, which can then be transformed into more complex SPLs (SM, HexCer, LacCer, GM3). As shown in Figure 4A, cinobufagin treatment increased the levels of 31 ceramides (Cer). Ceramides, the central hub of neurolipid metabolism and neutral lipids of sphingolipid metabolism, are potent tumor suppressors that regulate the processes of cell proliferation, differentiation, senescence, and apoptosis, and have attracted significant attention in the combination therapy of cancer. Krautbauer’s study demonstrates that Cer is significantly reduced in HCC tissues (Krautbauer et al., 2016). Drugs that modulate Cer-related enzymes are of increasing interest by which Cer levels can be increased, leading to autophagy and inhibition of tumor growth. It is believed that short-chain Cers can prevent tumor invasion, and according to the published reports, nanoliposome-loaded short-chain Cers are indeed effective against HCC (Li et al., 2018). Studies have also shown that Cers induce apoptosis (Liu et al., 2011). Cer is the main substance that induces mitochondrial outer membrane permeabilization (MOMP), and MOMP is a key step in apoptosis. Low Cer correlates with tumor progression and poor prognosis (Chang et al., 2015). However, there is limited literature on the association between hexosyl ceramide and cancer.

As shown in Figure 4B, cinobufagin treatment increased the levels of all identified eight monosialodihexosylganglioside (GM3). Among gangliosides, GM3 is found in the human body most frequently and has the most basic structure, consisting of three simple sugars: glucose, galactose, and sialic acid (Lipina and Hundal, 2015). Through its modulation of tyrosine kinase growth factor receptors, GM3 plays a significant role in controlling the proliferation, invasion, and metastasis of tumor cells (Zheng et al., 2019). A study has developed GM3 analogs, and through cytotoxicity assays and wound healing tests, the data suggests that GM3 analogs exhibit anticancer activity, which provides new ideas for finding new anti-tumor drugs for cancer treatment (Zheng et al., 2020). Studies have shown that KAI1 and GM3 act synergistically to inhibit cancer cell motility (Jung et al., 2016). Li et al. have found that GM3 and lyso-GM3 inhibit the migration of melanoma cells, and exhibit excellent antitumor activity (Li T. et al., 2021). The upregulation of GM3 levels in liver cancer cells may be responsible for the anti-hepatoma effects of cinobufagin.

As shown in Figure 4C, cinobufagin treatment increased the levels of 39 sphingomyelins (SM). Changes in sphingolipid metabolism have previously been associated with a variety of cancers, such as HCC, colorectal cancer, acute lymphoblastic leukemia, and squamous cell carcinoma of the head and neck. SM is the most abundant class of sphingolipids in the cell, an essential element of the cell membrane, and a key participant in cell function. In the cellular sphingolipid cycle, SM is hydrolyzed by SM enzymes to ceramides, which synthesize SM from scratch and participate in cell proliferation, growth, and apoptosis (Bienias et al., 2016). Relevant report suggests that SM enhances *in vivo* anti-tumor effects in colon tumor models. Cancer cells have lower levels of sphingomyelin (SM) than non-tumorigenic cells. SM appears to interact in a synergistic manner with apoptosis-inducing drugs, thereby enhancing their antitumor effects. Tumor formation is inhibited and survival is increased in mice by SM intake, and food SM supplementation lead to a decrease in colonic inflammation as well as colorectal cancer (Mazzei et al., 2011). Moreover, studies have shown that probiotic and SM blends are highly effective against pre-tumor lesions (Marzo et al., 2022). Huang et al. have performed a LC-MS-based sphingolipid analysis of human lung adenocarcinoma cell line A549, and find that a significant decrease in the levels of 35 of SMs (Huang et al., 2018). These results provide clues for analyzing the role of sphingolipids in cancer, suggesting that increased levels of SMs may be partially participant in the anti-tumor effects of cinobufagin.

#### 3.3.3 Glycerophospholipids

Glycerophospholipids are the most common phospholipids, mainly including Phosphatidylcholine (PC), Phosphatidylglycerol (PG), Phosphatidic acid (PA), Phosphatidylserine (PS), Phosphatidylethanolamine (PE), Phosphatidylinositol (PI), Cardiolipin (CL), Hemibismonoacylglycerophosphate (HBMP), Phosphatidylethanol (PEtOH), and Bis(monoacylglycero)phosphates (BMP). The effect of cinobufagin on class of lipids is shown in Table 2.

**TABLE 2:** The effect of cinobufagin on Glycerophospholipids.

Class	subClass	Number
Glycerophospholipids	Phosphatidylethanol (PEtOH)	Down (0), Up (1)
	Phosphatidylcholine (PC)	Down (97), Up (152)
	Phosphatidylethanolamine (PE)	Down (67), Up (28)
	Phosphatidylserine (PS)	Down (29), Up (28)
	Bis(monoacylglycero)phosphate (BMP)	Down (0), Up (1)
	Hemibismonoacylglycerophosphate (HBMP)	Down (0), Up (6)
	Phosphatidylglycerol (PG)	Down (23), Up (21)
	Phosphatidylinositol (PI)	Down (24), Up (18)
	Phosphatidic acid (PA)	Down (5), Up (17)
	Cardiolipin (CL)	Down (14), Up (0)

**FIGURE 5:**
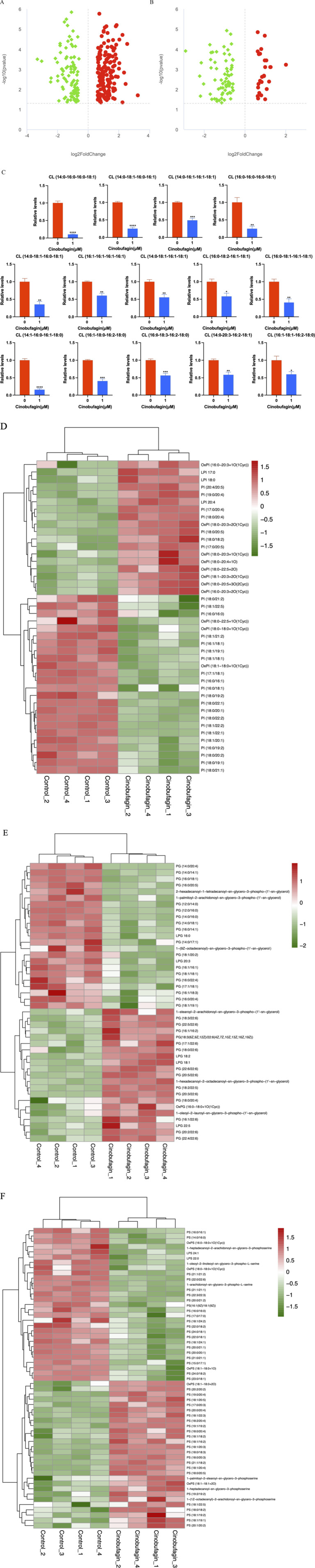
Graph analysis of the identified Glycerophospholipids in HepG2 cells treated with or without cinobufagin. **(A)** Volcano plot of the PC in HepG2 cells treated with or without cinobufagin. **(B)** Volcano plot of the PE in HepG2 cells treated with or without cinobufagin. Red circles represent upregulated lipids and green represents downregulated lipids. **(C)** Relative levels of the CL in HepG2 cells treated with or without cinobufagin. ^*^ *P* < 0.05, ^**^ *P* < 0.01, ^***^ *P* < 0.001, ^****^ *P* < 0.0001. **(D)** Heatmap analysis of the PI in HepG2 cells treated with or without cinobufagin. **(E)** Heatmap analysis of the PG in HepG2 cells treated with or without cinobufagin. **(F)** Heatmap analysis of the PS in HepG2 cells treated with or without cinobufagin. Red represents high level and green represents low level. Each row represents a single metabolite, each column represents one sample.

As shown in Figure 5A, cinobufagin treatment decreased the levels of 97 Phosphatidylcholines (PCs), detailed data are provided in Supplementary Table S1. Zhang X et al. have found that exercise decreases hepatic palmitate load and PC (18:1/18:1) levels, thereby reestablishing a microenvironment unfavorable to HCC (Zhang et al., 2024). Tumor cell proliferation in HCC is associated with increased *de novo* synthesis of PC via the Kennedy pathway (Hall et al., 2021). Marien et al. have used a targeted phospholipidomics platform based on shotgun mass spectrometry to identify 108 phospholipid species in non-small cell lung cancer and normal tissues, of which the most prominent changes are the increase of certain PCs in tumor tissues (Marien et al., 2015). Human malignant colon tissues have higher amounts of PCs because of their higher expression and decreased turnover (Dueck et al., 1996).

As shown in Figure 5B, cinobufagin treatment decreased the level of 67 phosphatidylethanolamines (PEs), detailed data are provided in Supplementary Table S2. PEs are the second most abundant phospholipids in mammalian cell membranes. It has been known that PE is elevated in several cancers. A study has shown that PE is exposed to the luminal surface of the tumor vascular endothelium. PE seems to be a general marker present in several solid cancer mice models, including spontaneous tumors (Stafford and Thorpe, 2011). In a serum metabolomics study of HCC, many glycerophospholipids, especially lysophos-phatidylethanolamine (LPE) and PE, were found to have significantly increased levels in HCC cases. Rashid et al. have found that PE and LPE are significantly upregulated in HCC cases compared to cirrhosis (Rashid et al., 2023).

As shown in Figure 5C, cinobufagin treatment decreased the level of all identified 14 cardiolipins (CL), a mitochondria-specific phospholipid that accounts for approximately 20% of the phospholipid mass of the mitochondrial membrane (IMM). Numerous investigations have revealed alterations in the composition and/or content of CL in cancer cells or tumor tissues. Zhong H et al. have claimed that HCC cell lines show an increase in saturated and monounsaturated CL compared to noncancerous cells (Zhong et al., 2017). Studies have noted variations in the concentration of several lipids including an increased level of CL in human prostate cancer tissues (Randall et al., 2019). High CL level and altered composition of the acyl chain have also been observed in thyroid tumors and have been suggested as tumor biomarkers. Studies have demonstrated that higher CL content and acyl chain modifications in liver mitochondria in a rat model of peritoneal carcinoma-induced malignant hypoxia of cancer, accompanied by reduced efficiency of oxidative phosphorylation and increased energy wastage (Dumas et al., 2011). The reduction of CL levels in liver cancer cells may be responsible for the anti-hepatoma effects of cinobufagin.

As shown in Figure 5D, cinobufagin treatment decreased the level of 24 PIs. PIs are enriched in tumor tissues. Compared with benign epithelial cells, Goto T et al. have identified significantly increased levels of PI (18:0/18:1), PI (18:0/20:3), and PI (18:0/20:2) in prostate cancer tissues by tandem mass spectrometry (Goto et al., 2014). Published reports have found PI (18:0/20:3) as a potential marker for various populations of breast cancer cells, and the increase of this lipid in breast cancer cells could explain their invasive capabilities (Kawashima et al., 2013).

As shown in Figure 5E, cinobufagin treatment decreased the level of 23 phosphatidylglycerols (PGs). It has been shown that polyunsaturated phosphatidylglycerols can promote esophageal cancer, studies have elucidated the reprogramming of phospholipid metabolism in esophageal adenocarcinoma and found that esophageal adenocarcinoma samples had a higher abundance of PGs compared with healthy esophageal epithelial tissue (Abbassi-Ghadi et al., 2020).

As shown in Figure 5F, cinobufagin treatment decreased the level of 29 phosphatidylserines (PSs), PSs are phospholipids located in the inner leaflet of the plasma membrane, which are activated after apoptosis and externalized after physiological stress. In the tumor microenvironment, exposure of PS to tumor cells and immune cells leads to immunosuppression and promotion of tumor growth (Hernández-Alvarez et al., 2019).

In summary, glycerophospholipids generally show high level in cancer and promote tumor progression through multiple mechanisms, and the downregulation of glycerophospholipids may be partially responsible for the anti-tumor effect of cinobufagin.

#### 3.3.4 Sterol lipids

**FIGURE 6:**
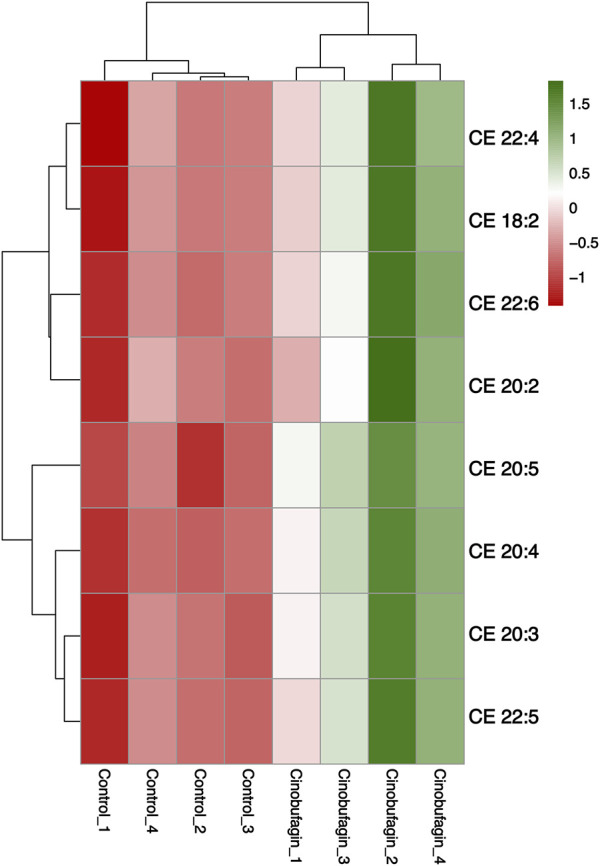
Heatmap analysis of the CE in HepG2 cells treated with or without cinobufagin. Red represents high level and green represents low level. Each row represents a single metabolite, each column represents one sample.

As shown in Figure 6, all eight identified cholesteryl esters (CEs) were elevated after cinobufagin treatment. Cholesteryl esters (CE), the combination products of cholesterol and fatty acids, are a storage form of cholesterol in the body and play an important role in maintaining intracellular free cholesterol homeostasis. Tumor cells maintain rapid proliferation by taking up large amounts of free cholesterol or hydrolyzing cholesteryl esters (Shen et al., 2016). High cholesterol levels improve post-surgical survival and reduce disease recurrence. Yang et al. have found that in HCC, cholesterol significantly inhibits cancer cell migration and invasion. CD44 is a glycoprotein that can control cancer metastasis, and cholesterol inhibits the invasive ability of HCC cells by virtue of blocking CD44-Ezrin interactions outside of rafts (Yang et al., 2018). Porphyrin cholesteryl ester is a type of CE, and studies have shown that porphyrin cholesteryl ester significantly reduces the fluidity of cancer cell membranes and inhibits the growth of cancer cells.

#### 3.3.5 Glycerolipids

**FIGURE 7:**
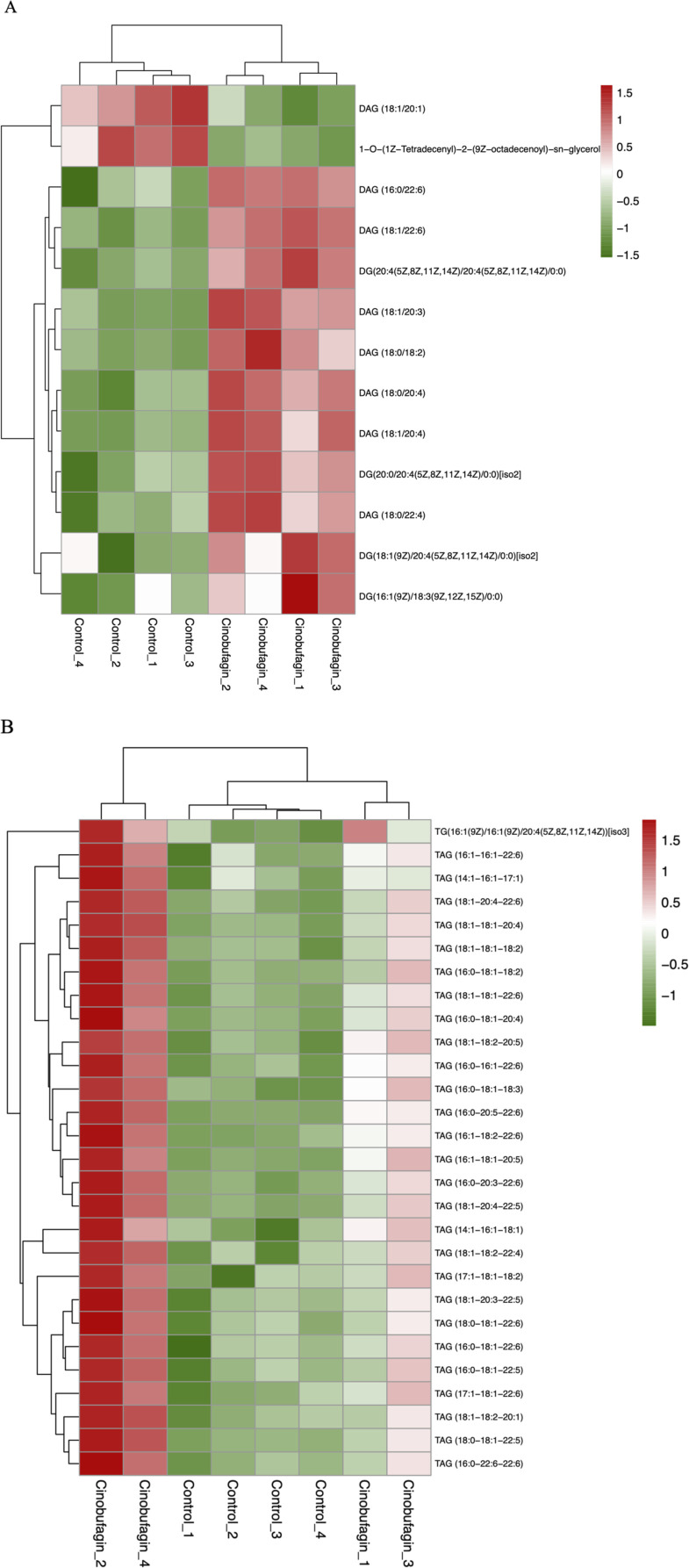
Graph analysis of the identified glycerolipids in HepG2 cells treated with or without cinobufagin. **(A)** Heatmap analysis of the DAG in HepG2 cells treated with or without cinobufagin. **(B)** Heatmap analysis of the TAG in HepG2 cells treated with or without cinobufagin. Red represents high level and green represents low level. Each row represents a single metabolite, each column represents one sample.

As shown in Figures 7A,B, cinobufagin treatment increased the level of all identified 28 triacylglycerols (TAG) and 11 diacylglycerols (DAG).

Deswal et al. demonstrated that TAG exerts a tumor-suppressive role in ovarian cancer by regulating lipid metabolism. Downregulation of lipid droplet-associated hydrolase (LDAH) enhances adipose triglyceride lipase (ATGL)-mediated TAG hydrolysis and promotes tumor growth and chemoresistance. Clinical evidence supports this mechanism, as a low TAG storage phenotype is associated with poor patient prognosis (Deswal et al., 2025). Hepatic lipidomics of HCC has been studied and interestingly, TAG with more than two double bonds (except 56:5 and 56:4 TAG) is significantly downregulated (Li et al., 2017). Elevated DAG expression may be a result of the toxic effects of cinobufagin on cancer cells, and a stress response may be reduced in the cells, and this stress response may include a disturbance in lipid metabolism, which leads to elevated DAG levels. Moreover, in response to cinobufagin, cancer cells may face disturbances in energy metabolism, and in order to maintain cell survival and proliferation, the cells may increase TAG storage to cope with possible energy demands.

### 3.4 Integration of transcriptomics and targeted lipid metabolomics analysis

We used the MetaboAnalyst database to perform a combined transcriptome and metabolomics joint pathway analysis of differential lipid metabolites and related differential genes. The results showed that the pathways associated with fatty acid metabolism (including biosynthesis of unsaturated fatty acids, fatty acid biosynthesis, fatty acid degradation, and fatty acid elongation), sphingolipid metabolism (including sphingolipid metabolism, glycosphingolipid biosynthesis-globo and isoglobo series, glycosphingolipid biosynthesis-lacto and neolacto series, glycosphingolipid biosynthesis-ganglio series), glycerophospholipid metabolism (including glycerophospholipid metabolism, ether lipid metabolism, glycosylphosphatidylinositol (GPI)-anchor biosynthesis) were correlated with the integrated metabolomic-transcriptomic analysis results. Green represented downregulated genes or metabolites, and red represented upregulated genes or metabolites, integrating transcriptomics and metabolomic analysis has a good insight to study the anti-hepatoma effects of cinobufagin. Detailed data are provided in Supplementary Table S3.

#### 3.4.1 Cinobufagin interferes with fatty acid metabolism in human HCC cells

**FIGURE 8:**
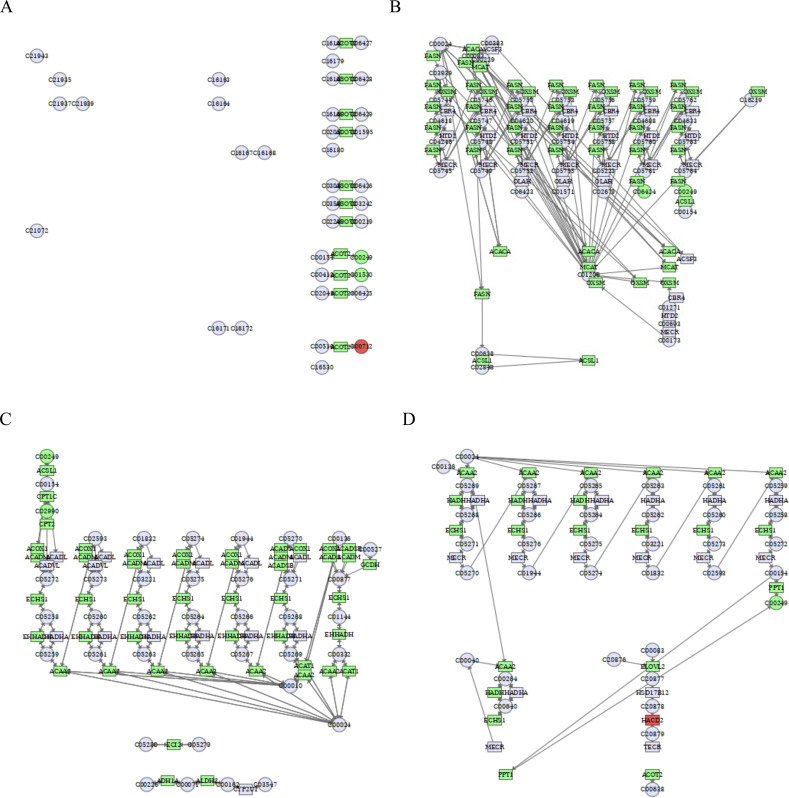
Integrated transcriptomics and metabolomics analyses enrich the pathways related to fatty acid metabolism. **(A)** Biosynthesis of unsaturated fatty acids. C00249: palmitic acid, C00712: oleic acid, C01530: stearic acid. **(B)** Fatty acid biosynthesis. C06424: myristic acid. **(C)** Fatty acid degradation. C02990: palmitoylcarnitine. **(D)** Fatty acid elongation. Green square or circle represented downregulated genes or metabolites, and red square or circle represented upregulated genes or metabolites.

Our previous study has demonstrated that transcriptomics data of human hepatoma cells exposed to cinobufagin identifies 672 metabolism-related differential genes (DEGs), and through untargeted metabolomics-transcriptomics integration analysis, we imply cinobufagin interferes with metabolic reprogramming, which comprises lipid, amino acid, nucleotide, and carbohydrate metabolism (Yang et al., 2023). Based on this, in our present study, we focused on analyzing the effects of cinobufagin on lipid metabolism (including fatty acid metabolism, sphingolipid metabolism, glycerophospholipid metabolism) using lipid-targeted metabolomics, and conducted a more complete study of the mechanism of action, which provide a theoretical basis of cinobufagin for treating HCC. Table 3 showed the DEGs associated with fatty acid metabolism analyzed by transcriptomic results, we performed a combined transcriptome and metabolomics joint pathway analysis of above differential genes and fatty acyl class differential metabolites. As shown in Figure 8A, and gene abundance of acyl-CoA thioesterase 2 (ACOT2) was inhibited in the pathway associated with biosynthesis of unsaturated fatty acids, more importantly, as a result, the stearic acid and palmitic acids were downregulated. Fatty acid metabolism plays an essential function in malignancies. First and foremost, tumor cells gain energy by increasing fat metabolism, and tumor cell proliferation can be sustained by a high amount of energy molecules such as fats, carbohydrates, and proteins. Tumor cells, on the other hand, improve the fluidity and stability of cell membranes by modifying the composition of cell membrane lipids, such as increasing the concentration of unsaturated fatty acids, allowing tumor cells to migrate and invade more easily. To address the biosynthetic demands of tumor growth, cancer cells frequently synthesize fatty acids (Svensson et al., 2016). ACOT2 is an enzyme involved in fatty acid metabolism, ACOT2 is crucial for fatty acid *β*-oxidation, it can catalyze the hydrolysis of oleoyl-CoA or palmitoyl-CoA, producing free oleic acid or palmitic acid. Overexpression of ACOT2 has been found to regulate prostaglandin production in breast cancer cells (Maloberti et al., 2010). Downregulation of ACOT2 in response to cinobufagin may affect the production of stearic acid and palmitic acid, thereby reducing their content.

**TABLE 3:** Fatty acyl class differential genes in transcriptome data.

Gene (name)	Down/Up	Gene (name)	Down/Up
HADHB	Down	ACOT4	Down
TTLL1	Down	ACSL4	Down
ACADS	Down	ELOVL6	Down
CPT1B	Down	CPT2	Down
ADH5	Down	OXSM	Down
GAREM2	Down	ALDH3A2	Down
ACSL1	Down	NBPF11	Down
ALDH2	Down	ACAA1	Down
ELOVL1	Down	FASN	Down
GCDH	Down	FOCAD	Down
PECR	Down	FADS2	Down
EHHADH	Down	ACAT2	Down
ALDH7A1	Down	SCD	Down
ACACA	Down	ECI2	Down
HADH	Down	MCAT	Down
FBF1	Down	ACADM	Down
ABHD17C	Down	ALDH1B1	Down
ACAA2	Down	ACADSB	Down
FADS1	Down	C1orf123	Down
ECHS1	Down	HACD3	Up
ACACB	Down	HACD2	Up
ACOX3	Down	IKZF5	Up
PPT2	Down	ABHD17B	Up

As shown in Figure 8B, cinobufagin inhibited the gene abundance of fatty acid synthase (FASN), 3-Oxoacyl-ACP synthase, mitochondrial (OXSM), acetyl-CoA carboxylase alpha (ACACA), malonyl-CoA-acyl carrier protein transacylase (MCAT), and acyl-CoA synthetase long-chain 1 (ACSL1). Many lipids are formed from fatty acids, which can be created by *de novo* biosynthesis. Increased amounts of fatty acids in tumor cells accelerate tumor growth (Currie et al., 2013). FASN is a key regulator of lipid metabolism that reprograms tumor cells to satisfy their high energy demands (Fhu and Ali, 2020). ACACA catalyzes the carboxylation of acetyl coenzyme A into malonyl coenzyme A, which is a rate-limiting step in fatty acid synthesis. Its overexpression can promote tumourigenesis and development. ACSL1, a member of the long-chain lipoyl-coenzyme A synthetase family (ACSLs), oversees activating long-chain fatty acids and can catalyze the acylation of ATP-dependent fatty acids to acyl-coenzyme A, which is the initial step in fatty acid production and entry into the cell. ACSL1 is overexpressed in cancer tissues, and inhibition of ACSL1 has been demonstrated to limit xenograft tumor growth *in vivo* (Ma et al., 2021). OXSM and MCAT are also key enzymes in fatty acid synthesis. During fatty acid synthesis, FASN is a key enzyme in the *de novo* synthesis of long-chain saturated fatty acid palmitic acid and myristic acid, and ACSL1 also converts palmitic acid to palmitoyl coenzyme A, which promotes protein palmitoylation and tumorigenesis (Peng et al., 2024). In Figure 8B, the inhibition of the level of palmitic acid and myristic acid may be induced by the suppression of FASN in response to cinobufagin.

As shown in Figure 8C, cinobufagin inhibited carnitine palmitoyltransferase 1C (CPT1C), carnitine palmitoyltransferase 2 (CPT2), ACSL1, acyl-CoA dehydrogenase, medium chain (ACADM), enoyl-CoA hydratase, short chain 1 (ECHS1), enoyl-CoA hydratase and 3-hydroxyacyl CoA-dehydrogenase (EHHADH), acetyl-CoA acyltransferase 2 (ACAA2), acyl-CoA dehydrogenase, short/branched chain (ACADSB) gene abundance, and more importantly, the level of metabolite palmitoylcarnitin and palmitic acid were suppressed in response to cinobufagin. Palmitoylcarnitine is a product of the CPT1-catalyzed reaction, while the activity of CPT2 is directly dependent on the concentration of palmitoylcarnitine. CPT1 is mostly present in liver cell membranes and consists of three isoforms, CPT1A, CPT1B, and CPT1C. Fatty acid *β*-oxidation is the main process of fatty acid degradation. These enzymes play important roles in fatty acid *β*-oxidation. CPT1 transfers carnitine lipoyl groups to mitochondria, converting them to coenzyme A and facilitating fatty acid *β*-oxidation. CPT2 is located in the inner membrane of mitochondria and transfers carnitine moieties from fatty acid Coenzyme A, allowing fatty acids to undergo further *β*-oxidation metabolism. CPT2 has a synergistic effect with CPT1 on fatty acid *β*-oxidation (Schlaepfer and Joshi, 2020). ACSL1 can catalyze the synthesis of palmitoyl coenzyme A from palmitic acid. Because palmitoyl coenzyme A is necessary for fatty acids to enter the *β*-oxidation pathway, ACSL1 activity affects palmitic acid metabolism. In specific contexts, palmitoyl-CoA is converted by CPT1 into palmitoylcarnitine, which enters the mitochondria for β-oxidation, providing energy and biosynthetic precursors to fuel cancer cell proliferation and progression. We also found that ACADM, ECHS1, EHHADH, and ACAA2 were upstream and downstream linked in several branched pathways, and they are all involved in the fatty acid *β*-oxidation process. During the degradation of medium/short-chain fatty acids, acyl-coenzyme A generates trans-2-enoyl-CoA through the dehydrogenation process of ACADM, which is followed by the hydration process of ECHS1 to generate L-3-Hydroxyacyl-CoA, and then by the dehydrogenation process of HADH to generate 3-Ketoacyl-CoA, and finally acetyl-coenzyme A through the ACAA2 thiolysis process to complete mitochondrial *β*-oxidation and provide energy to the cell. EHHADH is a bifunctional key enzyme in the fatty acid β-oxidation process, and its activity influences fatty acid oxidation.

As shown in Figure 8D, cinobufagin inhibited the gene abundance of acetyl-CoA acyltransferase 2 (ACAA2), hydroxyacyl-CoA dehydrogenase (HADH), ECHS1, palmitoyl-protein thioesterase 1 (PPT1), elongation of very long chain fatty acids protein 2 (ELOVL2), and acyl-CoA thioesterase 2 (ACOT2). ELOVL2 is an important member of the fatty acid elongase family and is closely related to the fatty acid elongation pathway. ELOVL2 lengthens the chain length of fatty acids by adding two carbon atoms to the fatty acid carbon chain. Aberrant overexpression of ELOVL2 may affect energy metabolism and biomolecule synthesis in tumor cells. A study have found that high expression of seven ELOVLs are observed in Renal cell carcinoma (RCC), and higher levels of ELOVL2 are associated with an increased incidence of poor patient prognosis, ELOVL2 promotes tumor progression by inhibiting apoptosis in renal cell carcinoma (Tanaka et al., 2022). PPT1 is a depalmitoylating enzyme that removes palmitoylation modifications from proteins, which in turn affects protein function. PPT deficiency leads to disruption of palmitic acid metabolism. PPT1 is not directly involved in fatty acid elongation, but palmitoylation modifications may indirectly affect fatty acid metabolism (Hellsten et al., 1996), this may have some effect on the metabolism of palmitic acid. These all results suggest that intervention in fatty acid metabolism may be responsible for the suppression of growth of human HCC cells induced by cinobufagin.

#### 3.4.2 Cinobufagin interferes with sphingolipid metabolism in human HCC cells

**FIGURE 9:**
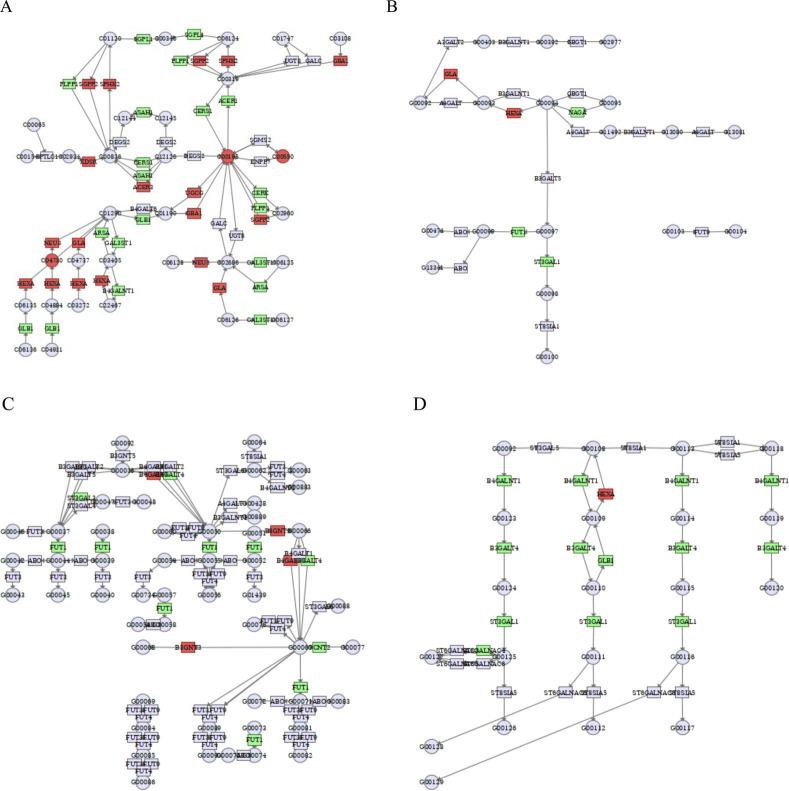
Integrated transcriptomics and metabolomics analyses enrich the pathways related to sphingolipid metabolism. **(A)** Sphingolipid metabolism. C00550: sphingomyelin, C00195: ceramide, C04730: GM3. **(B)** Glycosphingolipid biosynthesis-globo and isoglobo series. **(C)** Glycosphingolipid biosynthesis-lacto and neolacto series. **(D)** Glycosphingolipid biosynthesis-ganglio series. Green square or circle represented downregulated genes or metabolites, and red square or circle represented upregulated genes or metabolites.

As shown in Figure 9A and Table 4, by analyzing sphingolipid metabolic pathways, the key enzymes phospholipid phosphatase 1 (PLPP1), sphingosine-1-phosphate lyase 1 (SGPL1), N-acylsphingosine amidohydrolase 1 (ASAH1), ceramide synthase 1 (CERS1), galactosidase beta 1 (GLB1), alkaline ceramidase 1 (ACER1), ceramide kinase (CERK), arylsulfatase A (ARSA), galactose-3-O-sulfotransferase 1 (GAL3ST1), B4GALNT1 (Beta-1,4-N-acetylgalactosaminyltransferase 1) were inhibited by the effect of cinobufagin, and the key enzymes sphingosine-1-phosphate phosphatase 2 (SGPP2), sphingosine kinase 2 (SPHK2), 3-ketodihydrosphingosine reductase (KDSR), alkaline ceramidase 3 (ACER3), UDP-glucose ceramide glucosyltransferase (UGCG), glucosylceramidase beta 1 (GBA1), neuraminidase 3 (NEU3), HEXA (hexosaminidase subunit Alpha) and galactosidase alpha (GLA) were promoted by the effect of cinobufagin. Here we broadly divide them into two categories, key enzymes affecting sphingolipid synthesis and key enzymes for sphingolipid degradation. More importantly, the level of metabolite sphingomyelin, GM3, and ceramide were upregulated induced by cinobufagin. In the metabolomics section we demonstrated a result that sphingolipids (including Cer, SM, GM3) were almost all upregulated after cinobufagin treatment, and through the results of transcriptomics-metabolomics integrated analysis, we identified the key enzymes of sphingolipid metabolism were significantly regulated, which may be responsible for the increased level of sphingolipids. Sphingomyelin (SM) has anti-tumor effects. Cancer cells have lower levels of SM than non-tumor cells. SM intake in mice inhibits tumor formation and improves survival, and dietary SM supplementation reduces colonic inflammation and colorectal cancer. KDSR, an enzyme essential for sphingolipid synthesis from scratch, catalyzes the conversion of 3-ketodihydrosphingosine (KDS) to dihydrosphingosine (DHS) on cytoplasmic leaflets of the endoplasmic reticulum, a process that is essential for sphingolipid synthesis (Kihara and Igarashi, 2004). Accumulation of KDSR in cancer disrupts ER structure and function, leading to proteotoxic stress and cell death (Liu et al., 2022). The upregulation of KDSR may be responsible for the increased level of metabolite sphingomyelin in response to cinobufagin. Hydrolysis of SM produces ceramide (Arana et al., 2010). Ceramides are biologically active lipids with important physiological functions and are essential in the sphingolipid metabolic pathway. Ceramidase is essential for the hydrolysis of ceramides. ASAH1, known as acid ceramidase, also known as N-acetylsphingosine hydrolase, catalyzes the hydrolysis of ceramides to benzoic acid and fatty acids. Lack of ASAH1 has been documented to increase ceramide levels, thereby increasing toxicity to melanoma cells and inhibiting melanoma proliferation (Realini et al., 2016). ACER1 is also involved in the hydrolysis of ceramides. ACER1 is highly expressed in the epidermis and catalyzes the hydrolysis of ultra-long-chain ceramides (Zhang Z. et al., 2023). CERK catalyzes the catabolism of ceramide into ceramide-1-phosphate (C1P), which promotes tumor cell proliferation (Custodia et al., 2021). And the downregulation of ASAH1, ACER1, and CERK may be responsible for the increased level of ceramide (Cer). UGCG catalyzes the generation of glucose ceramide (GlcCer) from ceramide and UDP-glucose, which is a precursor for complex sphingolipid synthesis. GBA can convert GlcCer into glucose and ceramide, thus promoting ceramide synthesis. GLA can also catalyze the formation of lactose ceramide from globotriacyl ceramide. All of these key enzymes are upregulated in the pathway, which may be responsible for the increased level of Cer. GLB1 causes gangliosides to degrade in different situations, and the downregulation of GLB1 may be responsible for the increased level of ganglioside GM3 in response to cinobufagin. HEXA is a lysosomal enzyme that, together with the *β*-subunit (HEXB), forms *β*-hexosaminidase. GM3 is a simple ganglioside, a constituent of lipid rafts in cell membranes. HEXA catalyzes the hydrolysis of GM2 to GM3. The increased level of GM3 may be induced by upregulation of HEXA in response to cinobufagin. And GM3 has a good anti-tumor effect, GM3 blocks proliferative signaling in tumor cells by inhibiting integrin-induced EGFR phosphorylation (Wang et al., 2003), GM3 inhibits tumor angiogenesis by directly binding to VEGFR-2 (Chung et al., 2009).

**TABLE 4:** Sphingolipid differential genes in transcriptome data.

Gene (name)	Down/Up	Gene (name)	Down/Up
PLD2	Down	PIK3CB	Down
GAB2	Down	PPP2R5A	Down
TP53	Down	PLCB2	Down
PLD1	Down	PPP2R5B	Down
TSPAN1	Down	PIK3CD	Down
PPP2R2B	Down	S1PR5	Down
GAL3ST1	Down	ST3GAL2	Down
ASAH1	Down	SDCCAG8	Down
ARSA	Down	ROCK1	Down
MAP3K5	Down	MAPK12	Down
PRKCZ	Down	RAC2	Down
PLPP1	Down	TRADD	Down
PQLC3	Down	B4GALNT1	Down
PCNX3	Down	MAPK9	Down
B4GALT4	Down	MAPK3	Down
B3GALT4	Down	GLB1	Down
CERS4	Down	RAC3	Down
B3GNT3	Down	RASIP1	Down
NAGA	Down	AKT1	Down
TNFRSF1A	Down	FAIM	Down
MAPK11	Down	GNAI2	Down
S1PR2	Down	S1PR3	Down
PPP2R5D	Down	FUT1	Down
PRKCB	Down	AMZ1	Down
GNA12	Down	MTX1	Down
PPP2R3B	Down	GBA2	Down
ROCK2	Down	GCNT2	Down
ADORA1	Down	MAPK13	Down
ST6GALNAC4	Down	FYN	Down
NSMAF	Down	SGPP1	Up
BDKRB2	Down	B3GNT2	Up
PPP2R5C	Down	PPP2CB	Up
ASAH2B	Down	MAPK1	Up
B3GNT4	Down	UGCG	Up
PIK3R3	Down	SPHK1	Up
ST3GAL3	Down	NEU1	Up
SGPL1	Down	MAPK8	Up
PIK3R2	Down	PPP2CA	Up
CERK	Down	HEXB	Up
PPP2R1B	Up	B4GALT3	Up
GNAQ	Up	ACER3	Up
NRAS	Up	TNF	Up
GBA	Up	GLA	Up
CERS2	Up	PPP2R3C	Up
KDSR	Up	PRKCE	Up

As shown in Figure 9B, the key enzymes GLA, hexosaminidase subunit alpha (HEXA), N-acetylgalactosaminidase alpha (NAGA), fucosyltransferase 1 (FUT1), ST3 beta-galactoside alpha-2,3-sialyltransferase 1 (ST3GAL1) are closely related to glycosphingolipid biosynthesis-globo and isoglobo series. Glycosphingolipids are important components of cell membranes and its overexpression are common in cancer. Glycosphingolipids are composed of ceramides with glycan chains, including neutral and acidic glycosphingolipids. NAGA hydrolyzes α-conjugated N-acetylgalactosamine (*α*-GalNAc) residues in glycosphingolipids or glycoproteins, removing abnormally modified intermediates and preventing blockage of the glycosphingolipid synthesis pathway. FUT1 catalyzes the addition of α-1,2-fucose (Fuc) to the end of the glycan chain. ST3GAL1 adds α-2,3-linked sialic acid (Sia) to *β*-galactose (Gal) at the end of the glycan chain, which is involved in ganglioside synthesis and regulates cancer cell adhesion and signaling. The downregulation of NAGA, FUT1, and ST3GAL1 in response to cinobufagin may interfere with the glycosphingolipid synthesis to restrain the tumor growth.

As shown in Figure 9C, the key enzymes FUT1, ST3 beta-galactoside alpha-2,3-sialyltransferase 3 (ST3GAL3), beta-1,3-N-acetylglucosaminyltransferase 3 (B3GNT3), glucosaminyl (N-acetyl) transferase 2 (GCNT2) are closely related to glycosphingolipid biosynthesis-lacto and neolacto series. FUT1 functions in the synthesis of lacto-series and neolacto-series glycosphingolipids by modifying the glycan chain structure with the addition of fucose, α-1,2-fucose Fuc to the terminal galactose (Gal) of both types of glycan chains to form the H antigen. Overexpression of fucosylated GSLs promotes tumor cell metastasis and immune escape. In lacto-series and neolacto-series synthesis, FUT1 synergizes with B3GNT3 to accomplish glycan chain modification. The suppression of FUT1 in response to cinobufagin may interfere with the glycosphingolipid synthesis to restrain the tumor growth.

As shown in Figure 9D, the key enzymes beta-1,4-N-acetylgalactosaminyltransferase 1 (B4GALNT1), beta-1,3-galactosyltransferase 4 (B3GALT4), galactosidase beta 1 (GLB1), ST3 beta-galactoside alpha-2,3-sialyltransferase 1 (ST3GAL1) are closely related to glycosphingolipid biosynthesis-ganglio series. ST3GAL1, B4GALNT1, and B3GALT4 are key glycosyltransferases essential for glycosphingolipid synthesis. The downregulation of B4GALNT1, B3GALT4, GLB1, ST3GAL1 induced by cinobufagin may interfere with the glycosphingolipid synthesis to restrain the tumor growth. All these results suggest that cinobufagin may inhibit the growth of human HCC cells by affecting sphingolipid metabolic processes.

#### 3.4.3 Cinobufagin interferes with glycerophospholipid metabolism in HCC cells

**FIGURE 10:**
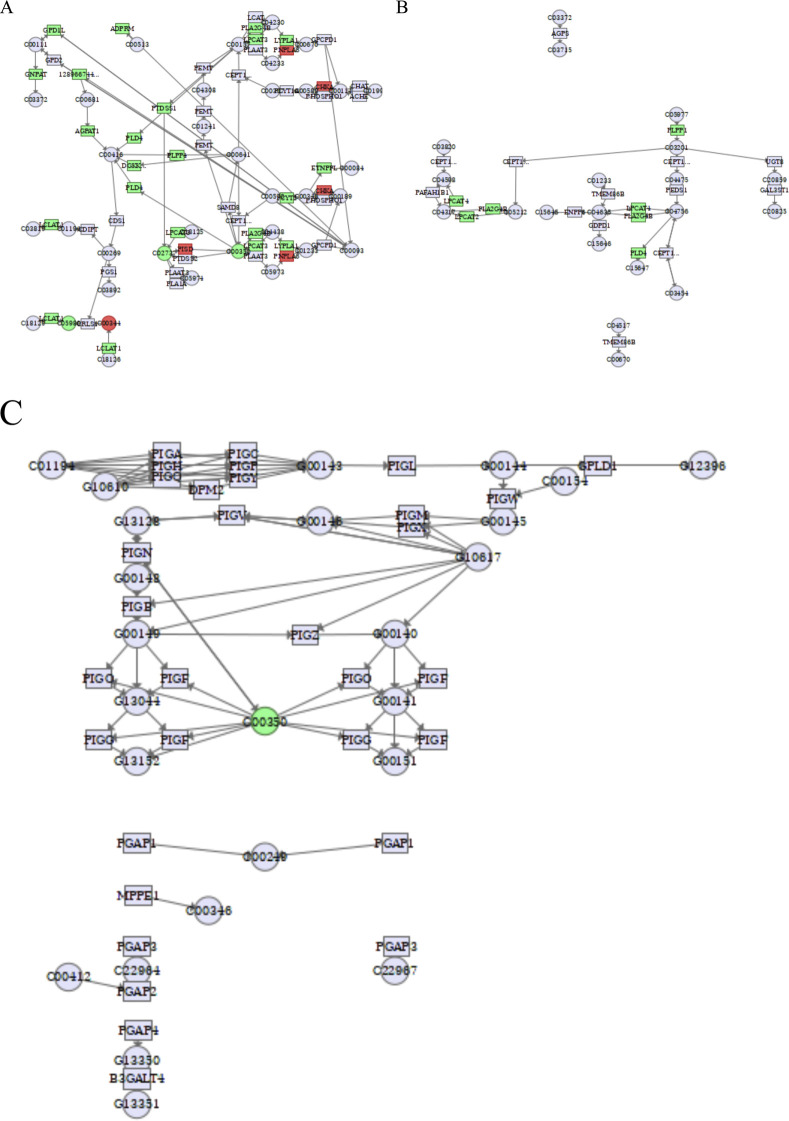
Integrated transcriptomics and metabolomics analyses enrich the pathways related to glycerophospholipid metabolism. **(A)**Glycerophospholipid metabolism. C05980: cardiolipin, C00344: phosphatidylglycerol, C02737: phosphatidylserine, C00350: phosphatidylethanolamine. **(B) **Ether lipid metabolism. **(C)** Glycosylphosphatidylinositol (GPI)-anchor biosynthesis. C00350: phosphatidylethanolamine. Green square or circle represented downregulated genes or metabolites, and red square or circle represented upregulated genes or metabolites.

There is a close relationship between glycerophospholipid metabolism and cancer, and changes in glycerophospholipid metabolites can be used as metabolic markers of cancer, including PE, PG, PS, and CL, which are involved in constituting cell membranes, energy metabolism, and signal transduction. The related genes that regulate glycerophospholipid metabolic processes also collectively influence and participate in the development of cancer. As shown in Figure 10 and Table 5, by analyzing the glycerophospholipid metabolism pathway, the key enzymes glycerol-3-phosphate dehydrogenase 1-like (GPD1L), ADP-ribose pyrophosphatase, mitochondrial (ADPRM), glyceronephosphate O-acyltransferase (GNPAT), lysocardiolipin acyltransferase 1 (LCLAT1), 1-acylglycerol-3-phosphate O-acyltransferase 1 (AGPAT1), phospholipase D4 (PLD4), diacylglycerol kinase kappa (DGKK), phosphatidylserine synthase 1 (PTDSS1), phospholipid phosphatase 4 (PLPP4), phospholipase A2 group IVB (PLA2G4B), lysophosphatidylcholine acyltransferase 3 (LPCAT3), LPCAT2, LPCAT4, and ethanolaminephosphotransferase 1-like (ETNPPL) were inhibited by cinobufagin, while the key enzymes phosphatidylserine decarboxylase (PISD), patatin-like phospholipase domain-containing 6 (PNPLA6), and choline kinase alpha (CHKA) were promoted by cinobufagin. More importantly, the level of metabolite cardiolipin, phosphatidylserine, and phosphatidylethanolamine were suppressed in response to cinobufagin. As shown in Figure 10A, there is a close relationship between phosphatidylserine and PTDSS1, LPCAT3, PISD. PTDSS1 is the core enzyme of PS synthesis, and the suppression of PTDSS1 may be participant in the decreased level of phosphatidylserine induced by cinobufagin. LPCAT3 is the key enzyme of PS remodeling, PISD is the key enzyme converting PS to PE. PTDSS1, LPCAT3, and PISD work together to maintain the dynamic equilibrium of PS. PISD is important in the phospholipid biosynthesis pathway because it regulates the homeostasis of phosphatidylserine and phosphatidylethanolamine, which is essential for the maintenance of the normal structure and function of cell membranes (Xu et al., 2019). In cancer, PISD frequently has a tumor suppressive function. In breast cancer cells, increasing PISD reduces primary tumor growth while also causing mitochondrial fragmentation, loss of mitochondrial mass, and disruption of cellular metabolism (Chen et al., 2018). And the upregulation of PISD may be responsible for the decreased level of phosphatidylserine in response to cinobufagin. LPCAT3, a member of the LPLAT family, plays an important role in the glycerophospholipid metabolism pathway and affects a variety of cellular functions. LPCAT3 is primarily responsible for regulating the abundance of PC species and generates PC by catalyzing LPC. In this process, LPCAT3 determines the composition and properties of membrane phospholipids by introducing specific fatty acid chains. LPCAT3 plays an important role in the onset and progression of many diseases such as intestinal tumors. LPCAT3 expression is elevated in many cancers and is associated with poor prognosis in cancers such as low-grade glioma (LGG), ovarian cancer (OV) and uveal melanoma (UVM) (Ke et al., 2022). And the inhibition of LPCAT3 may be responsible for the decreased level of PC. LCLAT1 is important for the functional maturation of cardiolipin (CL), a process that involves the incorporation of PUFAs to form biologically active PUFA-CL. Mitochondrial function of CL is impaired in the absence of LCLAT1. The decreased level of cardiolipin may be induced by the downregulation of LCLAT1 in response to cinobufagin. There are also enzymes that affect glycerophospholipid synthesis, GPAT catalyzes glycerol-3-phosphate to generate lysophosphatidic acid (LPA), AGPAT1 catalyzes LPA to generate phosphatidic acid (PA), GNPAT catalyzes ether lipid synthesis. The suppression of these genes may be partially responsible for the decreased level of some kinds of glycerophospholipids in response to cinobufagin in liver cancer cells.

**TABLE 5:** Glycerophospholipid differential genes in transcriptome data.

Gene (name)	Down/Up	Gene (name)	Down/Up
PLD2	Down	MVP	Down
AGPAT5	Down	ADPRM	Down
ETNPPL	Down	AGPAT1	Down
PLD1	Down	LPIN1	Down
TAZ	Down	GPAM	Down
AGPAT3	Down	PNPLA6	Down
PLA2G3	Down	GPD1L	Down
PLA2G6	Down	C17orf67	Down
PLPP1	Down	LPCAT1	Down
PTDSS1	Down	HRASLS2	Down
FAHD2A	Down	MTERF3	Down
GPD1	Down	ETNK2	Down
GPAT4	Down	LPIN3	Down
DGKQ	Down	C1orf131	Down
PCYT2	Down	ETNK1	Up
MBOAT2	Down	PLA2G4C	Up
MBOAT1	Down	CHKA	Up
JMJD7-PLA2G4B	Down	PISD	Up
LPCAT4	Down	ADGRF3	Up
GNPAT	Down	LYPLA2	Up
DGKA	Down	PLA2G4B	Up

As shown in Figure 10B, LPCAT2 and LPCAT4 were suppressed induced by cinobufagin. LPCAT2 plays an important role in ether lipid metabolism and is involved in the generation of acetylated phospholipids. LPCAT2 is highly expressed in a variety of tumors, and it has been shown that LPCAT2 enhances the malignant phenotype of tumor cells. LPCAT2 is also involved in the generation of lipid droplets, which reduces the efficacy of chemotherapeutic agents (Wang and Tontonoz, 2019). Published report has revealed that LPCAT4 is significantly highly expressed in HCC, which is positively correlated with the degree of infiltration of immune-infiltrating cells in the tumor microenvironment (Lu et al., 2023). Cinobufagin may inhibit the ether lipid metabolic pathway by down-regulating LPCAT2 and LPCAT4.

As shown in Figure 10C, on the cell surface of many eukaryotic organisms, proteins are tied to the plasma membrane via GPI anchors, which in turn affect membrane proteins. GPI-anchor dysregulation is regarded as a poor prognosis for cancer. High GPI-anchor biosynthesis leads to poor T cell status in the tumor microenvironment (TME) (Wu H. et al., 2024). GPI-anchors are glycolipids that are used to anchor proteins to the cell surface. The biosynthesis of GPI-anchors is a complex process involving the participation of several enzymes and genes. In this process, PE appears to be used as a donor substrate in an enzymatic reaction that affects GPI-anchor synthesis (Kinoshita and Fujita, 2016). And the decreased level of phosphatidylethanolamine may impair the GPI-anchor synthesis. The PI3K/Akt/mTOR signaling pathway is aberrant active in cancer, and its overactivation is thought to be a poor prognostic sign for cancer stem cells (CSCs) (Vivanco and Sawyers, 2002). These results suggest that cinobufagin may inhibit the growth of human HCC cells by affecting glycerophospholipid metabolism processes.

## 4 Discussion

Metabolomics uses analytical instrumentation combined with pattern recognition techniques to discover metabolic changes in subjects associated with disease states. Cancer alters cellular metabolism, and in parallel, metabolomics allows for early detection and diagnosis of cancer because it allows for rapid analysis of tissue or biofluid samples that complement patient genomic and proteomic information. Targeted lipid metabolomics, as a specialized subset of metabolomics, can be specifically analyzed to evaluate lipid change data. In addition, the combination of transcriptomics with metabolomics allows the construction of networks of differential metabolites and differential metabolism genes, which is a technological approach to analyze the mechanism of drug action. Cinobufagin has been shown in our previous study to reduce the proliferation and colony formation ability of human hepatoma cells *in vitro*. Moreover, cinobufagin induced G2 phase cell cycle arrest and DNA damage in cancer cells (Yang et al., 2022). In our present study, targeted lipid metabolomics combined with transcriptomics was performed on differential lipid compounds and lipid metabolism genes to further analyze the role of cinobufagin in exerting anti-hepatoma cell growth by interfering with lipid metabolic processes. The multi-omics data analysis brought us many hints to guide us to discover more anti-tumor action mechanisms of cinobufagin.

In this article, to investigate the mechanism of action of cinobufagin against the growth of human hepatoma cells, we performed targeted lipid metabolomics analysis on cancer cells treated with or without cinobufagin. After cinobufagin treatment, the total identification of metabolites in the positive ionic mode were 1,553, with 411 differential metabolites (DMEs), including 220 upregulated DMEs and 191 downregulated DMEs. In the negative ionic mode were 1,206, with 336 DMEs, including 198 upregulated DMEs and 138 downregulated DMEs. Our analysis results of targeted lipid metabolomics data indicated that DMEs involved glycerophospholipids, fatty acyls, sphingolipids, glycerolipids, saccharolipids, and sterol lipids in response to cinobufagin. Heatmaps were used to show changes in different classes of DMEs, and we found most levels of fatty acyl-related DMEs free fatty acids were decreased induced by cinobufagin, and the levels of fatty acyl-related DMEs acylcarnitine were all decreased after cinobufagin treatment. We identified that most levels of sphingolipid-related DMEs ceramides and sphingomyelines were increased after cinobufagin treatment, and the levels of sphingolipid-related DMEs monosialodihexosylganglioside were all increased in tumor cells after cinobufagin treatment. We identified that most levels of glycerophospholipid-related DMEs phosphatidylethanolamines were decreased after cinobufagin treatment, and the level of glycerophospholipid-related DMEs cardiolipin were all decreased after cinobufagin treatment. We identified the level of sterol lipid-related DMEs cholesteryl esters were all increased in tumor cells after cinobufagin treatment. We also found that most levels of glycerolipid-related DMEs diacylglycerols were increased after cinobufagin treatment, and the level of glycerolipid-related DMEs triacylglycerols were all increased after cinobufagin treatment. These results indicate that cinobufagin exhibits inhibition to liver cancer cell growth by interfering with fatty acyls, sphingolipids, glycerophospholipids, glycerolipids, and sterol lipids.

To further understand the molecular mechanism of cinobufagin against human hepatoma cell growth, we performed transcriptomic analysis of cinobufagin-treated and untreated tumor cells. 672 of differential genes were metabolism-related differential genes, 46 differential genes were related to fatty acid metabolism, 90 differential genes were related to sphingolipid metabolism, 42 differential genes were associated with glycerophospholipid metabolism. These results suggest that cinobufagin may affect fatty acid metabolism, sphingolipid metabolism, glycerophospholipid metabolism, which is consistent with the results of the metabolomics data above. Next, based on metabolomics combined with transcriptomics analysis, we demonstrated that intervention in fatty acid metabolism (including biosynthesis of unsaturated fatty acids, fatty acid biosynthesis, fatty acid degradation, and fatty acid elongation), sphingolipid metabolism (including sphingolipid metabolism, glycosphingolipid biosynthesis-globo and isoglobo series, glycosphingolipid biosynthesis-lacto and neolacto series, glycosphingolipid biosynthesis-ganglio series), and glycerophospholipid metabolism (including glycerophospholipid metabolism, ether lipid metabolism, glycosylphosphatidylinositol (GPI)-anchor biosynthesis) may be partially responsible for the effect of anti-hepatoma cell growth induced by cinobufagin. This study is of great significance for the application of cinobufagin and chansu in clinical liver cancer treatment and promotes the development of new drugs from traditional Chinese medicine in the field of antitumor. And in our future study, we will conduct in-depth studies on the anti-tumor mechanisms of cinobufagin indicated by the integrated omics analysis results.

Bufadienolides (such as bufalin, cinobufagin, arenobufagin, etc.) all utilize cholesterol as a precursor and are synthesized via a common biosynthetic pathway (Sousa et al., 2017). However, when intervening in lipid metabolism to exert antitumor effects, they exhibit both overlapping mechanisms and compound-specific unique targets and lipidomic signatures. Zhang et al. have demonstrated that bufadienolides may exert anti-prostate cancer effects by remodeling lipid metabolism. Integrated lipidomic and transcriptomic analyses revealed that these active components significantly downregulated long-chain lipid content in cancer cells and regulated multiple lipid metabolism-related genes, such as PLD1 (Zh et al., 2023). The combined treatment of cinobufagin and bufalin modulates sphingolipid and glycerophospholipid metabolism, which leads to mitochondrial-driven apoptosis and a systemic disruption of biomembranes within tumor cells (Zhang et al., 2021). Pang et al. have reported that bufalin suppresses the growth and liver metastasis of colorectal cancer by targeting the PI3K/AKT-mediated SREBP1/FASN pathway to inhibit *de novo* fatty acid synthesis (Pang et al., 2025). Published report have identified that bufalin suppresses triple-negative breast cancer by targeting the key lipid metabolic enzyme 2,4-dienoyl-CoA reductase (DECR1) to induce ferroptosis. Bufalin promotes DECR1 degradation via autophagy and ubiquitination pathways, leading to the inhibition of the SLC7A11/GPX4 axis and elevated markers of lipid peroxidation (Wu S. et al., 2024). Published report have reported that cinobufotalin suppresses HCC by directly inhibiting the lipid regulator SREBP1. Mechanistically, cinobufotalin interacts with SREBP1 to prevent its binding to sterol regulatory elements (SREs), thereby downregulating lipogenic enzymes and reducing the production of fatty acids. This inhibition of *de novo* lipogenesis ultimately leads to cell cycle arrest, apoptosis, and anti-tumor effects (Meng et al., 2021). Arenobufagin exerts anti-hepatoma effects by disrupting lipid homeostasis and interfering with amino acid supply. Arenobufagin significantly alters the levels of key lipids, notably glycerophospholipids (PCs, PEs), sphingolipids (SMs, Cer), and triglycerides (TGs), suppresses proteins involved in lipid metabolism, apoptosis, and autophagy, and activate SM synthase and arginine deiminase while inhibiting sphingomyelinase and the JAK-STAT3 signaling pathway (Zhao et al., 2020). In summary, bufadienolides exhibit diverse effects on lipid metabolism. Interfering with lipid metabolism is an important anti-tumor mechanism of this compound family. Our research further enrich the pharmacological profile of this natural product family, providing new insights for cancer treatment and significant implications for the development of lipid metabolism-targeted antitumor drugs.

In addition, although this study revealed that cinobufagin can exert anti-hepatoma effects by disrupting lipid metabolism, successfully translating it into clinical applications still faces challenges. It has problems such as poor water solubility, low oral bioavailability, and potential toxicity. Resolving these issues is crucial for its further development. In the future, we will explore advanced drug delivery strategies, such as developing HCC-targeted nanocarriers, improving water solubility and pharmacokinetic characteristics through rational prodrug design, enhancing solubility and stability, etc., to overcome these problems and promote their transformation from laboratory research to clinical applications.

Lipidomics data presented in the form of relative quantification cannot reflect the real lipid concentration. Furthermore, the study conducted in a single cell line has its limitations. In our subsequent studies, we will employ multiple cell models both to confirm the universality of the mechanism and to obtain precise concentration data through the absolute quantification of key lipids.

## Data Availability

The original contributions presented in the study are publicly available. This data can be found here: https://doi.org/10.6084/m9.figshare.31037137.
