# Supplementary material for: Targeted lipidomics meets transcriptomics: how cinobufagin rewires fatty acid, sphingolipid, and glycerophospholipid metabolism to combat hepatoma cell growth
Source: Front Pharmacol. 2026 Feb 9;16:1664915. doi: 10.3389/fphar.2025.1664915 (PMC12926493; doi:10.3389/fphar.2025.1664915)
Supplement: Supplementary file 2 [file Table1.docx]

**Table S1**

PC in HepG2 cells treated with or without cinobufagin.

| **Name** | **FC** | **log2FC** | **Pvalue** | **ROC** | **VIP** | **Up/Down** |
| --- | --- | --- | --- | --- | --- | --- |
| PC (18:2/22:5) | 2.00725859 | 1.00522649 | 1.3466E-05 | 1 | 1.34944396 | up |
| PC (15:0/20:4) | 4.77529685 | 2.25559042 | 1.9165E-05 | 1 | 1.35235682 | up |
| PC (18:1e/22:5) | 1.85054411 | 0.88794953 | 2.2503E-05 | 1 | 1.35809029 | up |
| PC (16:1e/20:5) | 1.79963159 | 0.8477016 | 7.2101E-05 | 1 | 1.35017932 | up |
| PC (16:1/20:5) | 3.32794871 | 1.7346332 | 9.1918E-05 | 1 | 1.35202252 | up |
| OxPC (16:0-18:2+2O) | 3.44886557 | 1.7861219 | 0.00018811 | 1 | 1.31165892 | up |
| PC(P-16:0/18:4(6Z,9Z,12Z,15Z)) | 2.42308644 | 1.27684587 | 0.00028907 | 1 | 1.34182975 | up |
| PC (16:1e/20:4) | 2.27055595 | 1.18304559 | 0.00032844 | 1 | 1.3332603 | up |
| PC (18:1/20:3) | 1.57869898 | 0.65873611 | 0.0004536 | 1 | 1.30747986 | up |
| PC (18:1e/20:5) | 2.70228904 | 1.434182 | 0.00047312 | 1 | 1.3402587 | up |
| PC (16:1/18:3) | 3.32062299 | 1.73145393 | 0.00066272 | 1 | 1.34985649 | up |
| OxPC (16:0-20:4+2O) | 3.07399148 | 1.62011317 | 0.00073621 | 1 | 1.30322208 | up |
| OxPC (18:1-18:2+2O) | 4.05319109 | 2.01905819 | 0.00093381 | 1 | 1.32186828 | up |
| LPC 20:4 | 1.58392046 | 0.66349989 | 0.00095174 | 1 | 1.26866121 | up |
| PC (18:5e/19:0) | 1.70802233 | 0.77232684 | 0.00097864 | 1 | 1.27706126 | up |
| PC (16:1e/16:0) | 1.72413755 | 0.78587488 | 0.00209259 | 1 | 1.29464627 | up |
| 1-lauroyl-sn-glycero-3-phosphocholine | 1.67210028 | 0.74166137 | 0.00237063 | 1 | 1.23527359 | up |
| OxPC (18:1-20:4+2O) | 3.81400205 | 1.93130562 | 0.00238312 | 1 | 1.30479654 | up |
| PC (17:1/18:3) | 2.46379431 | 1.30088181 | 0.00361698 | 1 | 1.2618478 | up |
| PC (16:0/16:3) | 1.69089535 | 0.75778738 | 0.00380978 | 1 | 1.28611109 | up |
| PC (20:4/22:5) | 1.80412344 | 0.85129806 | 0.00473099 | 1 | 1.27941414 | up |
| OxPC (18:1e/20:4+1O(1Cyc)) | 1.97227448 | 0.97986035 | 0.00486795 | 1 | 1.23386255 | up |
| PC (18:1e/22:6) | 2.58718839 | 1.37138511 | 0.00527272 | 1 | 1.28896255 | up |
| PC (18:5e/22:0) | 2.40240579 | 1.26447986 | 0.00686367 | 1 | 1.23418919 | up |
| PC (16:0/16:4) | 1.55770018 | 0.63941757 | 0.01309395 | 1 | 1.12541949 | up |
| PC (14:1e/16:0) | 2.22193702 | 1.15181792 | 0.01539768 | 1 | 1.16590106 | up |
| 1-tetradecyl-sn-glycero-3-phosphocholine | 2.02788691 | 1.0199772 | 0.02229361 | 1 | 1.08479763 | up |
| PC (16:0/16:2) | 2.3101196 | 1.20796755 | 0.02348023 | 1 | 1.17775353 | up |
| PC(14:1(9Z)/16:1(9Z)) | 2.15231833 | 1.10589147 | 0.02955608 | 0.9375 | 1.07781584 | up |
| OxPC (18:0-20:4+1O) | 1.74034807 | 0.79937587 | 0.03709642 | 0.875 | 1.0123613 | up |
| PC (20:5e/14:0) | 3.40700019 | 1.76850203 | 2.7795E-07 | 1 | 1.36884703 | up |
| PC (20:4/20:5) | 7.50216043 | 2.90730611 | 4.747E-07 | 1 | 1.36816154 | up |
| PC (14:0e/20:2) | 1.65271625 | 0.72483906 | 1.6938E-06 | 1 | 1.36339272 | up |
| PC (16:2e/18:4) | 3.63309539 | 1.86119925 | 6.3536E-06 | 1 | 1.35684264 | up |
| PC (17:1/17:1) | 2.28504813 | 1.19222455 | 7.1532E-06 | 1 | 1.36428112 | up |
| PC (20:4e/9:0) | 3.47650366 | 1.79763711 | 7.2237E-06 | 1 | 1.36470437 | up |
| PC (15:0/16:2) | 2.14696113 | 1.10229607 | 8.1242E-06 | 1 | 1.35536279 | up |
| PC (20:4/22:6) | 2.0034876 | 1.00251358 | 1.2198E-05 | 1 | 1.35737606 | up |
| PC (20:5/22:6) | 2.39933002 | 1.26263161 | 1.5488E-05 | 1 | 1.35175864 | up |
| LPC 20:3 | 4.12855373 | 2.04563648 | 3.3405E-05 | 1 | 1.37178841 | up |
| LysoPC(P-18:0) | 3.06772855 | 1.61717083 | 3.5443E-05 | 1 | 1.35426157 | up |
| PC (22:6e/13:1) | 2.48456217 | 1.31299164 | 3.8325E-05 | 1 | 1.34146776 | up |
| PC (22:5e/16:2) | 2.0474869 | 1.03385422 | 4.9589E-05 | 1 | 1.34419273 | up |
| PC (22:5e/13:0) | 5.84169854 | 2.54638791 | 5.4436E-05 | 1 | 1.35890939 | up |
| PC (17:0/20:5) | 3.58643387 | 1.84255003 | 6.1381E-05 | 1 | 1.36336616 | up |
| PC (15:0/18:3) | 2.86959213 | 1.52084569 | 7.595E-05 | 1 | 1.3391606 | up |
| LPC 16:2 | 2.39634978 | 1.26083851 | 7.9979E-05 | 1 | 1.33266695 | up |
| PC (14:0e/18:5) | 2.96672323 | 1.56887034 | 8.1242E-05 | 1 | 1.33189019 | up |
| PC (18:0/20:5) | 1.57340491 | 0.65388999 | 8.6256E-05 | 1 | 1.33120029 | up |
| PC (18:3e/13:1) | 3.20656085 | 1.68102678 | 8.9753E-05 | 1 | 1.33482724 | up |
| PC (16:0/17:2) | 1.74808759 | 0.80577748 | 0.00011393 | 1 | 1.32624384 | up |
| PC (20:5/22:5) | 3.14050154 | 1.65099498 | 0.00011863 | 1 | 1.33705079 | up |
| PC (17:1/16:2) | 2.21475873 | 1.14714955 | 0.00013956 | 1 | 1.35975174 | up |
| LPC 20:2 | 1.69971464 | 0.76529255 | 0.00014388 | 1 | 1.32713811 | up |
| LPC 19:0 | 1.65798634 | 0.72943212 | 0.00017928 | 1 | 1.32323998 | up |
| PC (16:2e/20:0) | 1.75089765 | 0.80809476 | 0.00019251 | 1 | 1.32312967 | up |
| PC (17:2/17:2) | 2.51063304 | 1.32805117 | 0.00019329 | 1 | 1.32975428 | up |
| PC (16:2e/20:5) | 2.67764637 | 1.42096544 | 0.00019869 | 1 | 1.34026757 | up |
| PC (17:1/20:4) | 2.11077178 | 1.0777706 | 0.00019948 | 1 | 1.35404236 | up |
| PC (18:4/18:4) | 2.55951385 | 1.35586981 | 0.00020695 | 1 | 1.35315563 | up |
| 1-O-palmitoyl-2-O-arachidonoyl-sn-glycero-3-phosphocholine | 3.05241431 | 1.6099508 | 0.00022742 | 1 | 1.31502292 | up |
| PC (18:1/20:5) | 1.61273878 | 0.68951278 | 0.0002453 | 1 | 1.33964356 | up |
| PC (16:1/20:4) | 2.59482329 | 1.37563629 | 0.00024639 | 1 | 1.32113093 | up |
| PC (15:0/16:1) | 1.54447425 | 0.62711582 | 0.00024892 | 1 | 1.36063632 | up |
| PC (16:1/22:5) | 1.70744201 | 0.77183658 | 0.00028398 | 1 | 1.3392658 | up |
| PC (22:6e/13:0) | 3.58050834 | 1.84016443 | 0.00028677 | 1 | 1.30818098 | up |
| PC (20:5/20:5) | 19.9248137 | 4.31649433 | 0.00028795 | 1 | 1.36142761 | up |
| LPC 17:1 | 1.63804867 | 0.71197823 | 0.00031776 | 1 | 1.34170111 | up |
| PC (18:0/20:3) | 1.56913223 | 0.64996693 | 0.00033635 | 1 | 1.3121449 | up |
| PC (18:2e/16:3) | 3.25690317 | 1.70350083 | 0.00039832 | 1 | 1.31748577 | up |
| PC (18:2/18:3) | 2.33533802 | 1.22363138 | 0.00041472 | 1 | 1.32800209 | up |
| PC (14:0e/18:3) | 2.80008346 | 1.48546983 | 0.00043823 | 1 | 1.32541294 | up |
| PC (15:0/22:6) | 2.13404863 | 1.09359305 | 0.00047209 | 1 | 1.34060884 | up |
| PC (20:5e/9:0) | 4.11451466 | 2.04072226 | 0.00049953 | 1 | 1.3424084 | up |
| LPC 22:4 | 1.64468862 | 0.71781447 | 0.00068866 | 1 | 1.30042166 | up |
| PC (20:0/22:6) | 2.12478333 | 1.08731573 | 0.00070889 | 1 | 1.28713355 | up |
| PC (16:2e/21:2) | 2.02365498 | 1.01696334 | 0.00082754 | 1 | 1.33066039 | up |
| PC (22:6e/9:0) | 2.51360748 | 1.32975938 | 0.00088596 | 1 | 1.2960574 | up |
| PC (16:2/16:3) | 2.41475591 | 1.27187736 | 0.00089013 | 1 | 1.27622613 | up |
| PC (14:0e/22:6) | 2.47842099 | 1.30942127 | 0.000942 | 1 | 1.32822542 | up |
| PC (18:4e/22:3) | 2.33732824 | 1.22486035 | 0.00096678 | 1 | 1.27575587 | up |
| PC (14:1e/20:2) | 1.81534973 | 0.86024752 | 0.00098709 | 1 | 1.27534288 | up |
| PC (18:1e/16:3) | 1.84306926 | 0.88211029 | 0.00107159 | 1 | 1.27352666 | up |
| PC (22:5e/9:0) | 2.78948461 | 1.47999859 | 0.00115441 | 1 | 1.28167158 | up |
| PC (17:1/18:2) | 2.8604526 | 1.51624344 | 0.00120528 | 1 | 1.26540042 | up |
| PC (16:2e/18:2) | 3.07597338 | 1.62104302 | 0.0012081 | 1 | 1.31819827 | up |
| PC (15:1/15:1) | 2.2032892 | 1.13965887 | 0.00121376 | 1 | 1.26570049 | up |
| PC (22:6e/10:0) | 2.02850458 | 1.02041656 | 0.00133953 | 1 | 1.26156728 | up |
| PC (18:5e/20:1) | 2.88601886 | 1.52908073 | 0.00142143 | 1 | 1.26914356 | up |
| PC (12:0/16:3) | 5.11128101 | 2.35368491 | 0.00145992 | 1 | 1.29791635 | up |
| PC (22:6e/20:4) | 2.73283771 | 1.45039979 | 0.00148192 | 1 | 1.28922679 | up |
| PC (18:4e/2:0) | 1.5394681 | 0.62243198 | 0.00149265 | 1 | 1.28703801 | up |
| PC (16:2/18:5) | 1.90827569 | 0.93226961 | 0.00149287 | 1 | 1.3131343 | up |
| LPC 20:5 | 1.59593772 | 0.67440436 | 0.00155959 | 1 | 1.32017971 | up |
| LPC 17:0 | 1.98823408 | 0.99148762 | 0.00162524 | 1 | 1.28947857 | up |
| PC (22:5e/16:0) | 1.71988448 | 0.78231167 | 0.00171809 | 1 | 1.26040216 | up |
| PC (14:1e/22:5) | 2.3274665 | 1.2187604 | 0.00188898 | 1 | 1.32684503 | up |
| PC (20:4e/17:2) | 1.99345929 | 0.99527414 | 0.00198164 | 1 | 1.24545014 | up |
| PC (18:0/19:0) | 2.80710881 | 1.48908499 | 0.00218004 | 1 | 1.28572991 | up |
| PC (22:4e/10:0) | 3.52202282 | 1.81640426 | 0.0023238 | 1 | 1.28966219 | up |
| PC (14:0e/24:4) | 1.65571808 | 0.72745704 | 0.00242411 | 1 | 1.24621992 | up |
| LysoPC(20:5(5Z,8Z,11Z,14Z,17Z)) | 1.69307522 | 0.75964607 | 0.00256001 | 1 | 1.25130308 | up |
| PC (22:6e/22:6) | 1.96366492 | 0.97354877 | 0.00256857 | 1 | 1.23517982 | up |
| PC (20:4/20:4) | 2.96057271 | 1.56587628 | 0.00265515 | 1 | 1.32675978 | up |
| PC (16:2/20:5) | 1.72046748 | 0.78280062 | 0.00279335 | 1 | 1.28707579 | up |
| PC (18:3e/16:4) | 2.41422795 | 1.2715619 | 0.00330874 | 1 | 1.28676549 | up |
| PC (20:0/20:4) | 2.63723881 | 1.39902822 | 0.00333053 | 1 | 1.28782261 | up |
| PC (19:2/20:4) | 1.95338942 | 0.96597959 | 0.00346802 | 1 | 1.23036985 | up |
| PC (15:0/16:4) | 2.5538992 | 1.35270158 | 0.00439765 | 1 | 1.24553947 | up |
| PC (14:0/18:3) | 2.7384155 | 1.45334137 | 0.00480705 | 1 | 1.26562037 | up |
| PC (10:0/11:0) | 1.76711336 | 0.82139459 | 0.00490155 | 1 | 1.23571024 | up |
| PC (18:3/18:3) | 3.27666655 | 1.71222886 | 0.00527593 | 1 | 1.22611713 | up |
| PC (22:5e/17:2) | 5.42056284 | 2.43844266 | 0.00531551 | 1 | 1.28952654 | up |
| PC (22:6e/22:4) | 2.56260079 | 1.35760875 | 0.00573645 | 1 | 1.18929669 | up |
| PC (19:2/18:5) | 1.70857209 | 0.77279112 | 0.0061249 | 1 | 1.20074539 | up |
| PC (16:1/18:5) | 1.97180646 | 0.97951795 | 0.00662487 | 1 | 1.28190179 | up |
| PC (20:5e/18:0) | 1.56784005 | 0.64877838 | 0.00680803 | 1 | 1.27313464 | up |
| PC (20:0/20:5) | 2.56626521 | 1.35967027 | 0.00760409 | 1 | 1.23699953 | up |
| PC (22:3e/20:3) | 1.98222666 | 0.98712194 | 0.00761838 | 1 | 1.21863542 | up |
| LPC 18:3 | 2.04328857 | 1.03089297 | 0.00767417 | 1 | 1.21955664 | up |
| PC (22:6e/20:5) | 2.03545891 | 1.0253541 | 0.00781684 | 1 | 1.17982082 | up |
| PC (17:0/18:4) | 3.0934715 | 1.62922674 | 0.00868431 | 1 | 1.23447225 | up |
| PC (17:0/20:4) | 3.31293509 | 1.72810994 | 0.00927935 | 1 | 1.25840322 | up |
| PC (22:6/22:6) | 1.73520544 | 0.79510648 | 0.00994578 | 1 | 1.23628284 | up |
| PC (22:3e/9:0) | 3.38657886 | 1.75982859 | 0.01049159 | 1 | 1.21149974 | up |
| PC (20:3/20:3) | 2.05502917 | 1.03915887 | 0.01083853 | 0.9375 | 1.14285775 | up |
| PC (14:0/20:5) | 2.07799704 | 1.0551936 | 0.01155491 | 1 | 1.22576544 | up |
| PC (10:0/16:3) | 2.55526917 | 1.35347527 | 0.01162453 | 1 | 1.17324888 | up |
| PC (19:0/20:4) | 3.11889075 | 1.64103302 | 0.01233829 | 1 | 1.17974528 | up |
| PC (13:0/16:4) | 2.256968 | 1.17438597 | 0.01363305 | 1 | 1.19889812 | up |
| PC (14:1e/16:4) | 2.57222977 | 1.36301952 | 0.01607534 | 1 | 1.21461214 | up |
| PC (19:1/20:5) | 1.71863553 | 0.78126363 | 0.01765344 | 1 | 1.14201945 | up |
| PC (20:4e/17:1) | 1.89635696 | 0.92323055 | 0.01886515 | 1 | 1.21173369 | up |
| PC (17:2/16:4) | 2.44208165 | 1.28811144 | 0.01910432 | 1 | 1.14518643 | up |
| PC (16:1/22:6) | 9.55514867 | 3.25627832 | 0.01950554 | 1 | 1.20534812 | up |
| PC (18:2e/26:4) | 1.55745899 | 0.63919417 | 0.02018761 | 0.9375 | 1.12721969 | up |
| PC (16:1/18:2) | 2.63840681 | 1.39966703 | 0.02033971 | 1 | 1.12474554 | up |
| 1-Palmitoyl-2-docosahexaenoyl-sn-glycero-3-phosphocholine | 1.74976162 | 0.80715839 | 0.02067912 | 1 | 1.08546791 | up |
| PC (20:2/22:6) | 2.04288235 | 1.03060612 | 0.02482542 | 1 | 1.10880907 | up |
| PC (17:0/18:1) | 1.61134681 | 0.68826704 | 0.02592047 | 1 | 1.06740489 | up |
| PC (22:6e/21:0) | 1.66189592 | 0.73283003 | 0.02687059 | 1 | 1.07602397 | up |
| PC (8:0/13:1) | 1.68823294 | 0.75551398 | 0.02734591 | 1 | 1.1254867 | up |
| PC (20:3/20:4) | 3.07807538 | 1.62202856 | 0.02753115 | 1 | 1.14202012 | up |
| PC (19:2/22:6) | 2.50941338 | 1.32735015 | 0.02914783 | 1 | 1.15326955 | up |
| PC (17:1/20:5) | 3.29203117 | 1.71897799 | 0.02962824 | 1 | 1.12714331 | up |
| PC (20:3e/22:5) | 1.53928876 | 0.6222639 | 0.03223263 | 0.9375 | 1.05622066 | up |
| PC (16:0e/26:4) | 2.31298695 | 1.20975713 | 0.03347114 | 1 | 1.11085756 | up |
| PC (22:3e/20:2) | 1.69175184 | 0.75851796 | 0.03704547 | 1 | 1.11861436 | up |
| PC (18:2e/20:5) | 1.71218874 | 0.77584174 | 0.04178327 | 0.9375 | 1.01510552 | up |
| PC (18:4e/26:4) | 1.7527458 | 0.80961678 | 0.0428571 | 1 | 1.05226398 | up |
| PC (5:0/16:4) | 1.7940076 | 0.84318601 | 0.04401105 | 0.9375 | 1.03016594 | up |
| PC (18:1e/25:0) | 4.76793818 | 2.25336553 | 0.04493244 | 1 | 1.09340517 | up |
| PC (16:1e/14:0) | 0.28388413 | -1.8166259 | 0.00014897 | 1 | 1.35191809 | down |
| OxPC (16:0-18:0+1O(1Cyc)) | 0.45816128 | -1.1260726 | 0.00096186 | 1 | 1.30828272 | down |
| PC (12:0/14:0) | 0.30626877 | -1.7071298 | 0.00097824 | 1 | 1.2815121 | down |
| 1-hexadecyl-2-hexadecanoyl-sn-glycero-3-phosphocholine | 0.53820564 | -0.8937706 | 0.00100783 | 1 | 1.33242791 | down |
| PC (16:0/18:0) | 0.62565785 | -0.6765542 | 0.001335 | 1 | 1.3290059 | down |
| PC (18:0/22:1) | 0.38044584 | -1.394237 | 0.00416843 | 1 | 1.26908471 | down |
| PC (18:1e/18:2) | 0.56583856 | -0.8215376 | 0.0084295 | 1 | 1.25669012 | down |
| PC (16:1e/16:2) | 0.50738861 | -0.978837 | 0.01609758 | 1 | 1.19866515 | down |
| OxPC (18:1-18:0+1O(1Cyc)) | 0.5980978 | -0.7415467 | 0.02145293 | 0.9375 | 1.10404369 | down |
| OxPC (18:0-18:0+1O(1Cyc)) | 0.64073175 | -0.6422076 | 0.03781802 | 1 | 1.08113613 | down |
| 1-[(11Z,14Z)]-icosadienoyl-sn-glycero-3-phosphocholine | 0.46929739 | -1.0914256 | 1.4464E-06 | 1 | 1.37280525 | down |
| PC (14:0/14:0) | 0.37565111 | -1.4125347 | 3.2456E-06 | 1 | 1.36671786 | down |
| PC (13:0/13:0) | 0.34006335 | -1.5561246 | 4.4322E-06 | 1 | 1.36217136 | down |
| PC (16:0/16:0) | 0.39024461 | -1.3575494 | 6.4801E-06 | 1 | 1.37224913 | down |
| PC (14:0e/18:1) | 0.50754404 | -0.9783951 | 1.2574E-05 | 1 | 1.36483966 | down |
| LPC 24:1 | 0.42850528 | -1.2226151 | 2.3826E-05 | 1 | 1.35421782 | down |
| PC (15:1/16:1) | 0.62184557 | -0.6853718 | 2.4336E-05 | 1 | 1.34700802 | down |
| LPC 22:1 | 0.4689007 | -1.0926457 | 3.2946E-05 | 1 | 1.34514984 | down |
| PC (14:0e/16:1) | 0.40371689 | -1.3085842 | 3.9911E-05 | 1 | 1.36847045 | down |
| PC (18:5e/21:2) | 0.05351731 | -4.2238505 | 4.8612E-05 | 1 | 1.37126161 | down |
| PC (16:0e/14:0) | 0.27836242 | -1.8449636 | 5.3311E-05 | 1 | 1.36822885 | down |
| PC (19:2/18:3) | 0.46684515 | -1.098984 | 5.6366E-05 | 1 | 1.33681833 | down |
| PC (21:1/21:2) | 0.28053984 | -1.8337224 | 6.4373E-05 | 1 | 1.34015268 | down |
| PC (14:0/15:0) | 0.56101977 | -0.8338765 | 7.2354E-05 | 1 | 1.33944919 | down |
| LPC 14:0 | 0.49673424 | -1.0094539 | 7.3025E-05 | 1 | 1.34355338 | down |
| PC (14:0/16:0) | 0.36446516 | -1.4561472 | 8.4368E-05 | 1 | 1.3629558 | down |
| PC (18:0e/14:0) | 0.37954033 | -1.3976749 | 0.00023838 | 1 | 1.31803562 | down |
| PC (19:0/18:1) | 0.53577999 | -0.9002874 | 0.00023862 | 1 | 1.32825119 | down |
| PC (16:0/14:1) | 0.53024687 | -0.9152639 | 0.00025701 | 1 | 1.31538042 | down |
| PC (20:1/22:5) | 0.46002454 | -1.1202173 | 0.00027038 | 1 | 1.34783857 | down |
| PC (16:0e/16:1) | 0.36534504 | -1.4526685 | 0.00029086 | 1 | 1.33224474 | down |
| PC (14:1e/8:0) | 0.22756821 | -2.1356291 | 0.00029571 | 1 | 1.34769696 | down |
| LPC 21:1 | 0.663103 | -0.5926951 | 0.00030856 | 1 | 1.30660457 | down |
| PC (22:0/22:1) | 0.09957451 | -3.3280797 | 0.00031073 | 1 | 1.33133227 | down |
| PC (18:5/22:6) | 0.12105637 | -3.0462491 | 0.0003896 | 1 | 1.36033001 | down |
| LysoPC(22:2(13Z,16Z)) | 0.49508471 | -1.0142527 | 0.00044831 | 1 | 1.29824411 | down |
| PC (18:1/22:2) | 0.24758609 | -2.0139978 | 0.00045491 | 1 | 1.35549519 | down |
| PC (16:0e/18:0) | 0.52384189 | -0.9327967 | 0.00060819 | 1 | 1.31718285 | down |
| PC (14:0e/19:1) | 0.51499293 | -0.9573755 | 0.00064833 | 1 | 1.29091344 | down |
| 1-Myristoyl-2-linoleoyl-sn-glycero-3-phosphocholine | 0.51242059 | -0.9645997 | 0.00067229 | 1 | 1.29007336 | down |
| PC (20:1/22:6) | 0.45223501 | -1.1448554 | 0.00073218 | 1 | 1.34117773 | down |
| PC (15:0/15:0) | 0.39442907 | -1.3421622 | 0.00076195 | 1 | 1.28232069 | down |
| PC(o-16:1(9Z)/20:0) | 0.52333044 | -0.9342059 | 0.00076526 | 1 | 1.28973633 | down |
| PC (18:0e/10:0) | 0.56146879 | -0.8327223 | 0.00086786 | 1 | 1.32809359 | down |
| PC (14:1/14:1) | 0.37377122 | -1.4197726 | 0.00087107 | 1 | 1.30194514 | down |
| PC (16:0e/16:0) | 0.301079 | -1.731786 | 0.00096441 | 1 | 1.3512369 | down |
| 1-tetradecanoyl-2-[(15Z)-tetracosenoyl]-sn-glycero-3-phosphocholine | 0.32320015 | -1.6295002 | 0.00122462 | 1 | 1.26492721 | down |
| PC (15:1/17:2) | 0.50906995 | -0.9740642 | 0.00139841 | 1 | 1.26779863 | down |
| PC (14:0/16:1) | 0.46521563 | -1.1040285 | 0.00140638 | 1 | 1.29904054 | down |
| PC (20:0/20:0) | 0.31155372 | -1.6824472 | 0.0018068 | 1 | 1.2735995 | down |
| PC (18:0e/11:0) | 0.59883363 | -0.7397728 | 0.00196259 | 1 | 1.28975684 | down |
| PC (20:0/18:1) | 0.41515861 | -1.2682655 | 0.00207658 | 1 | 1.31509467 | down |
| PC (14:0e/8:0) | 0.25382599 | -1.9780883 | 0.0022106 | 1 | 1.30494899 | down |
| PC (20:0/20:1) | 0.18529459 | -2.4321073 | 0.00274386 | 1 | 1.29590152 | down |
| PC (18:1/21:1) | 0.33803358 | -1.5647615 | 0.00279253 | 1 | 1.25532915 | down |
| PC (22:1/22:6) | 0.354402 | -1.4965413 | 0.00312249 | 1 | 1.26339776 | down |
| PC (16:0/17:0) | 0.5261262 | -0.9265192 | 0.003629 | 1 | 1.23114691 | down |
| PC (15:0/15:1) | 0.6091734 | -0.7150751 | 0.00375849 | 1 | 1.23196681 | down |
| PC (20:0/21:2) | 0.25133465 | -1.9923185 | 0.00376866 | 1 | 1.28435964 | down |
| PC (20:2/21:2) | 0.50297027 | -0.991455 | 0.00409327 | 1 | 1.27917662 | down |
| PC (19:0/20:1) | 0.18437546 | -2.4392814 | 0.00441426 | 1 | 1.29845732 | down |
| 1-O-octadecyl-2-arachidonoyl-sn-glycero-3-phosphocholine | 0.48232985 | -1.051908 | 0.00492231 | 1 | 1.30182392 | down |
| PC (15:1/20:4) | 0.59652644 | -0.745342 | 0.0051587 | 1 | 1.28637592 | down |
| PC (17:0/19:1) | 0.46603794 | -1.1014807 | 0.00605364 | 1 | 1.26226363 | down |
| PC (22:4e/12:0) | 0.54983992 | -0.8629164 | 0.00613185 | 1 | 1.27144086 | down |
| LysoPC(24:1(15Z)) | 0.31489365 | -1.6670634 | 0.00761562 | 1 | 1.21113186 | down |
| PC (14:0/16:3) | 0.53991535 | -0.8891948 | 0.00769786 | 1 | 1.20822483 | down |
| PC (18:1e/13:1) | 0.61070233 | -0.7114587 | 0.00862269 | 1 | 1.24385285 | down |
| PC (22:0/18:1) | 0.32333229 | -1.6289105 | 0.00961188 | 1 | 1.22813966 | down |
| PC (14:0e/20:1) | 0.58455428 | -0.7745911 | 0.01029405 | 1 | 1.2330375 | down |
| PC (15:1/20:3) | 0.59362187 | -0.7523838 | 0.01058516 | 1 | 1.20635449 | down |
| PC (18:0e/8:0) | 0.63538241 | -0.6543029 | 0.01070892 | 1 | 1.19495596 | down |
| PC (14:1e/18:0) | 0.58126312 | -0.7827367 | 0.01164872 | 1 | 1.15937001 | down |
| PC (16:0/20:1) | 0.52846152 | -0.9201297 | 0.01173006 | 1 | 1.2255156 | down |
| PC (18:0e/13:0) | 0.51076636 | -0.9692646 | 0.01206094 | 0.9375 | 1.13452678 | down |
| 1-tetradecyl-2-hexadecanoyl-sn-glycero-3-phosphocholine | 0.57317119 | -0.802962 | 0.01450023 | 1 | 1.11625131 | down |
| PC (18:1e/16:0) | 0.63419335 | -0.6570054 | 0.01531971 | 1 | 1.19650707 | down |
| PC (16:0/19:2) | 0.60594637 | -0.722738 | 0.01609449 | 1 | 1.15062074 | down |
| PC (18:1/20:1) | 0.4589536 | -1.1235798 | 0.01620229 | 1 | 1.19974813 | down |
| PC (18:0/18:0) | 0.52755939 | -0.9225946 | 0.01638826 | 1 | 1.13891873 | down |
| PC (18:2e/22:0) | 0.60300498 | -0.7297582 | 0.01719811 | 1 | 1.1687281 | down |
| PC(P-18:1(9Z)/22:2(13Z,16Z)) | 0.62986218 | -0.6668919 | 0.02107175 | 1 | 1.19861743 | down |
| PC (21:2/20:3) | 0.49048991 | -1.0277046 | 0.02242419 | 1 | 1.08035944 | down |
| PC (14:0e/17:0) | 0.46341223 | -1.109632 | 0.02282723 | 1 | 1.17437452 | down |
| PC (15:0/16:3) | 0.48029632 | -1.0580033 | 0.02398902 | 1 | 1.15881849 | down |
| PC (12:0/12:0) | 0.36076227 | -1.4708796 | 0.02507034 | 1 | 1.15999099 | down |
| PC (18:1e/17:0) | 0.52949242 | -0.9173181 | 0.02884942 | 1 | 1.06792926 | down |
| PC (22:3/22:3) | 0.34094511 | -1.5523886 | 0.02922263 | 1 | 1.1535517 | down |
| PC (14:1e/27:0) | 0.59812162 | -0.7414892 | 0.03153217 | 1 | 1.12262005 | down |
| PC (19:0/19:0) | 0.45169673 | -1.1465736 | 0.03390223 | 1 | 1.1114969 | down |
| PC (18:2e/21:0) | 0.39903061 | -1.3254287 | 0.03393678 | 1 | 1.10020592 | down |
| PC (15:1/22:5) | 0.39941737 | -1.324031 | 0.03444665 | 1 | 1.12508501 | down |
| PC (22:2/22:3) | 0.5350997 | -0.9021204 | 0.03463396 | 0.9375 | 1.08589445 | down |
| PC (22:1/22:2) | 0.26551096 | -1.9131567 | 0.03660435 | 1 | 1.11801718 | down |
| PC (8:0/9:0) | 0.57002957 | -0.8108913 | 0.04180927 | 1 | 1.09503524 | down |
| PC (14:0/15:1) | 0.58521166 | -0.7729696 | 0.04231438 | 1 | 1.08441324 | down |
| PC (16:0e/17:0) | 0.3858167 | -1.3740125 | 0.04333087 | 1 | 1.05150752 | down |

**Table S2**

PE in HepG2 cells treated with or without cinobufagin.

| **Name** | **FC** | **log2FC** | **Pvalue** | | **ROC** | **VIP** | **Up/Down** |
| --- | --- | --- | --- | --- | --- | --- | --- |
| PE (20:5/20:5) | 8.47776658 | 3.08368425 | | 7.094E-07 | 1 | 1.36161222 | up |
| PE (18:2e/20:4) | 1.54855922 | 0.63092655 | | 1.4137E-05 | 1 | 1.3474105 | up |
| PE (20:1/20:5) | 1.95741794 | 0.96895183 | | 2.1501E-05 | 1 | 1.35099846 | up |
| PE (18:2e/20:5) | 1.7396356 | 0.79878514 | | 9.7068E-05 | 1 | 1.3433968 | up |
| PE (14:1e/20:4) | 1.67422704 | 0.74349518 | | 0.0001659 | 1 | 1.33673143 | up |
| PE (20:1/20:3) | 1.60668524 | 0.68408732 | | 0.00017864 | 1 | 1.32224962 | up |
| PE (18:2/20:5) | 2.07291293 | 1.05165952 | | 0.00036245 | 1 | 1.31533947 | up |
| OxPE (18:0-20:4+2O) | 1.96375205 | 0.97361278 | | 0.00036978 | 1 | 1.31061284 | up |
| PE (20:4/20:5) | 4.04223454 | 2.01515303 | | 0.00058583 | 1 | 1.32770823 | up |
| PE (20:3/20:5) | 2.35019742 | 1.23278195 | | 0.00076588 | 1 | 1.34574026 | up |
| PE (18:1e/20:5) | 1.90459725 | 0.92948596 | | 0.00209993 | 1 | 1.31427115 | up |
| PE (18:2e/22:5) | 1.90457684 | 0.9294705 | | 0.00218133 | 1 | 1.24272145 | up |
| PE (16:0/16:2) | 1.67785062 | 0.74661428 | | 0.00375165 | 1 | 1.25425321 | up |
| PE (18:3e/20:4) | 2.27157096 | 1.18369037 | | 0.00411863 | 1 | 1.27043189 | up |
| OxPE (18:1-18:1+2O) | 1.76597649 | 0.82046614 | | 0.00426463 | 1 | 1.21043788 | up |
| PE (18:0e/22:5) | 2.09674948 | 1.0681545 | | 0.0099241 | 1 | 1.2378324 | up |
| PE (20:1/18:2) | 1.63369299 | 0.70813689 | | 0.01543038 | 1 | 1.22016308 | up |
| OxPE (18:0-20:4+4O(2Cyc)) | 1.73443666 | 0.79446716 | | 0.01995866 | 1 | 1.1500173 | up |
| OxPE (18:0-20:3+4O(1Cyc)) | 1.57016851 | 0.65091939 | | 0.02900543 | 1 | 1.1017402 | up |
| PE (18:1e/20:4) | 1.9941596 | 0.99578088 | | 0.03107064 | 0.875 | 1.04127905 | up |
| 1-(hexadec-1-enyl)-2-hexadecanoyl-sn-glycero-3-phosphoethanolamine | 16.5023123 | 4.04459628 | | 0.03422488 | 0.9375 | 1.10340915 | up |
| PE (18:4e/20:2) | 1.53954662 | 0.62250556 | | 0.00012868 | 1 | 1.32422647 | up |
| PE (16:2e/20:4) | 2.40271542 | 1.26466578 | | 0.000781 | 1 | 1.33742543 | up |
| PE (20:4/20:4) | 1.97473767 | 0.98166102 | | 0.00078582 | 1 | 1.30740647 | up |
| PE (18:5e/22:2) | 1.78946433 | 0.83952779 | | 0.00237779 | 1 | 1.23736107 | up |
| PE (18:0e/20:5) | 1.58377471 | 0.66336713 | | 0.00545457 | 1 | 1.19483361 | up |
| PE (18:4e/22:2) | 1.56216799 | 0.64354961 | | 0.00815047 | 1 | 1.17017349 | up |
| PE (20:3e/20:3) | 1.54290766 | 0.62565172 | | 0.02208521 | 1 | 1.13546306 | up |
| PE (16:0/18:1) | 0.53656613 | -0.8981721 | | 1.1696E-05 | 1 | 1.34708251 | down |
| PE (16:0/20:5) | 0.61581534 | -0.6994303 | | 1.8065E-05 | 1 | 1.34710376 | down |
| PE (16:0/16:0) | 0.30454241 | -1.7152849 | | 5.8893E-05 | 1 | 1.35586414 | down |
| PE (16:0e/16:0) | 0.48829862 | -1.0341644 | | 7.5779E-05 | 1 | 1.33914974 | down |
| PE (18:1e/14:0) | 0.31533607 | -1.6650379 | | 0.00014651 | 1 | 1.35156592 | down |
| PE (16:1e/14:0) | 0.49637667 | -1.0104928 | | 0.00016936 | 1 | 1.33492123 | down |
| LPE 16:1 | 0.58885521 | -0.7640151 | | 0.00018358 | 1 | 1.31251799 | down |
| PE (17:0/16:1) | 0.65674083 | -0.6066039 | | 0.00032554 | 1 | 1.3180626 | down |
| PE (18:0e/16:0) | 0.31184442 | -1.6811016 | | 0.00033312 | 1 | 1.34382783 | down |
| PE (16:0/17:0) | 0.52117346 | -0.9401645 | | 0.0003776 | 1 | 1.30250645 | down |
| LPE 20:3 | 0.64236244 | -0.6385406 | | 0.00083281 | 1 | 1.31141891 | down |
| PE (18:0e/14:0) | 0.3167159 | -1.6587388 | | 0.00086461 | 1 | 1.32319317 | down |
| PE (14:0/14:0) | 0.53742609 | -0.8958617 | | 0.00094027 | 1 | 1.27435011 | down |
| PE (16:0/19:1) | 0.6244769 | -0.6792799 | | 0.00100239 | 1 | 1.27249226 | down |
| PE (16:1/20:3) | 0.55492722 | -0.8496295 | | 0.00195413 | 1 | 1.30369657 | down |
| PE (20:1/22:1) | 0.21371117 | -2.2262658 | | 0.00223516 | 1 | 1.32004889 | down |
| PE (14:0/17:0) | 0.35872496 | -1.47905 | | 0.00345006 | 1 | 1.23447963 | down |
| PE (16:1/16:1) | 0.44554594 | -1.1663539 | | 0.00346178 | 1 | 1.29045576 | down |
| PE (22:5e/14:1) | 0.5104882 | -0.9700505 | | 0.00407972 | 1 | 1.26983044 | down |
| PE (18:0/22:6) | 0.56950186 | -0.8122275 | | 0.00415105 | 1 | 1.26043489 | down |
| PE (16:0/18:0) | 0.57623708 | -0.7952656 | | 0.0050326 | 1 | 1.25648734 | down |
| LPE 18:0 | 0.60953658 | -0.7142153 | | 0.00514088 | 1 | 1.22853803 | down |
| PE (18:1e/16:0) | 0.47035948 | -1.0881643 | | 0.00525734 | 1 | 1.2880016 | down |
| PE (20:1/22:6) | 0.52441763 | -0.9312119 | | 0.00623846 | 1 | 1.18129407 | down |
| PE (18:1/20:1) | 0.58385684 | -0.7763134 | | 0.00675999 | 1 | 1.23996487 | down |
| OxPE (18:0-18:2+2O) | 0.53290742 | -0.9080432 | | 0.00724713 | 1 | 1.25341103 | down |
| PE (17:0/18:1) | 0.64622454 | -0.6298926 | | 0.00809968 | 1 | 1.2027639 | down |
| PE (16:1/18:3) | 0.47381272 | -1.0776112 | | 0.00847577 | 1 | 1.20735426 | down |
| OxPE (18:1-18:0+1O) | 0.50910921 | -0.9739529 | | 0.01125784 | 1 | 1.23868916 | down |
| PE (16:0e/17:1) | 0.59376494 | -0.7520362 | | 0.01450625 | 1 | 1.15060637 | down |
| PE (16:1/17:1) | 0.61127522 | -0.710106 | | 0.01536837 | 1 | 1.17092208 | down |
| PE (16:1e/4:0) | 0.30797602 | -1.6991101 | | 0.01775906 | 1 | 1.1834819 | down |
| PE (18:0e/15:0) | 0.45844413 | -1.1251822 | | 0.01799104 | 1 | 1.18639576 | down |
| PE (4:0/18:0) | 0.35535437 | -1.4926696 | | 0.02974845 | 1 | 1.14166756 | down |
| PE (24:0/18:1) | 0.32271567 | -1.6316645 | | 0.03089641 | 1 | 1.13645879 | down |
| OxPE (16:0-18:0+1O(1Cyc)) | 0.49643098 | -1.0103349 | | 0.03471992 | 0.875 | 1.04512765 | down |
| PE (18:1e/4:0) | 0.38526392 | -1.376081 | | 0.03908661 | 1 | 1.09730331 | down |
| PE (16:0/20:4) | 0.63177391 | -0.6625197 | | 5.5187E-05 | 1 | 1.33726848 | down |
| PE (16:1/18:1) | 0.56154846 | -0.8325176 | | 0.00010696 | 1 | 1.33959739 | down |
| PE (18:3/20:5) | 0.13202418 | -2.9211259 | | 0.00012345 | 1 | 1.32492781 | down |
| PE (16:0e/14:1) | 0.38233346 | -1.3870966 | | 0.00013318 | 1 | 1.34106296 | down |
| PE (15:0/16:0) | 0.33494543 | -1.578002 | | 0.00014 | 1 | 1.32560357 | down |
| PE (14:0/20:5) | 0.66615997 | -0.5860594 | | 0.00035467 | 1 | 1.36000169 | down |
| 1-palmitoyl-2-oleoyl-sn-glycero-3-phosphoethanolamine | 0.35834148 | -1.480593 | | 0.00046369 | 1 | 1.34365562 | down |
| PE (14:0/16:0) | 0.22681868 | -2.1403886 | | 0.00048519 | 1 | 1.34107095 | down |
| PE (17:1/18:1) | 0.64313676 | -0.6368025 | | 0.00065355 | 1 | 1.30037227 | down |
| PE (16:0/16:1) | 0.37824685 | -1.4026 | | 0.00094931 | 1 | 1.33915957 | down |
| PE (18:0/18:1) | 0.55227848 | -0.8565322 | | 0.00127879 | 1 | 1.26671908 | down |
| PE (16:0e/18:1) | 0.50588646 | -0.9831145 | | 0.00177024 | 1 | 1.25192682 | down |
| PE (18:0/20:3) | 0.62937076 | -0.6680179 | | 0.00184745 | 1 | 1.30688156 | down |
| PE (19:2/18:3) | 0.58502999 | -0.7734175 | | 0.00209471 | 1 | 1.30373124 | down |
| PE (20:3/20:3) | 0.53377266 | -0.9057027 | | 0.00291382 | 1 | 1.29399132 | down |
| 1-oleoyl-2-linoleyl-sn-glycero-3-phosphoethanolamine | 0.56890756 | -0.8137339 | | 0.00515432 | 1 | 1.19861088 | down |
| PE (14:0e/20:0) | 0.66197839 | -0.595144 | | 0.00539515 | 1 | 1.19674164 | down |
| 1-Myristoleoyl-2-docosahexaenoyl-sn-glycero-3-phosphoethanolamine | 0.24652279 | -2.0202071 | | 0.00541899 | 1 | 1.22737726 | down |
| PE (14:0e/18:0) | 0.44003488 | -1.1843102 | | 0.00858419 | 1 | 1.1704237 | down |
| 1,2-Dimyristoyl-sn-glycero-3-phosphoethanolamine | 0.40643516 | -1.2989029 | | 0.00973286 | 1 | 1.18161734 | down |
| PE (14:0/16:1) | 0.66176044 | -0.595619 | | 0.01130262 | 1 | 1.19289989 | down |
| 1,2-Diarachidoyl-sn-glycero-3-phosphoethanolamine | 0.66260849 | -0.5937714 | | 0.01155221 | 1 | 1.18613959 | down |
| PE (20:3/22:6) | 0.46954014 | -1.0906796 | | 0.01543674 | 1 | 1.19104978 | down |
| PE (18:2/19:2) | 0.55296697 | -0.8547348 | | 0.01646813 | 1 | 1.14766507 | down |
| PE (18:1/24:1) | 0.21936122 | -2.1886196 | | 0.02138136 | 1 | 1.14055898 | down |
| PE (17:2/17:2) | 0.4504245 | -1.1506428 | | 0.02835858 | 0.9375 | 1.07347187 | down |
| PE (17:0/22:6) | 0.61697602 | -0.6967137 | | 0.03024231 | 0.9375 | 1.04282678 | down |
| PE (11:0/11:0) | 0.37906598 | -1.3994791 | | 0.0316919 | 1 | 1.14406794 | down |
| PE (22:1/22:6) | 0.41804274 | -1.2582776 | | 0.03538135 | 1 | 1.13372308 | down |
| PE (17:1/19:2) | 0.45344113 | -1.1410129 | | 0.04763127 | 1 | 1.09134472 | down |
